# Analysis and Characterization of Proteins Associated with Outer Membrane Vesicles Secreted by *Cronobacter* spp.

**DOI:** 10.3389/fmicb.2017.00134

**Published:** 2017-02-09

**Authors:** Mahendra H. Kothary, Gopal R. Gopinath, Jayanthi Gangiredla, Prasad V. Rallabhandi, Lisa M. Harrison, Qiong Q. Yan, Hannah R. Chase, Boram Lee, Eunbi Park, YeonJoo Yoo, Taejung Chung, Samantha B. Finkelstein, Flavia J. Negrete, Isha R. Patel, Laurenda Carter, Venugopal Sathyamoorthy, Séamus Fanning, Ben D. Tall

**Affiliations:** ^1^U. S. Food and Drug AdministrationLaurel, MD, USA; ^2^Centre for Food Safety, School of Public Health, Physiotherapy and Sports Science, WHO Collaborating Centre for Cronobacter, University College, DublinDublin, Ireland

**Keywords:** outer membrane vesicles, outer membrane proteins, EM, PCR, microarray

## Abstract

Little is known about secretion of outer membrane vesicles (OMVs) by *Cronobacter*. In this study, OMVs isolated from *Cronobacter sakazakii, Cronobacter turicensis*, and *Cronobacter malonaticus* were examined by electron microscopy (EM) and their associated outer membrane proteins (OMP) and genes were analyzed by SDS-PAGE, protein sequencing, BLAST, PCR, and DNA microarray. EM of stained cells revealed that the OMVs are secreted as pleomorphic micro-vesicles which cascade from the cell's surface. SDS-PAGE analysis identified protein bands with molecular weights of 18 kDa to >100 kDa which had homologies to OMPs such as GroEL; OmpA, C, E, F, and X; MipA proteins; conjugative plasmid transfer protein; and an outer membrane auto-transporter protein (OMATP). PCR analyses showed that most of the OMP genes were present in all seven *Cronobacter* species while a few genes (OMATP gene, *groEL, ompC, mipA, ctp*, and *ompX*) were absent in some phylogenetically-related species. Microarray analysis demonstrated sequence divergence among the OMP genes that was not captured by PCR. These results support previous findings that OmpA and OmpX may be involved in virulence of *Cronobacter*, and are packaged within secreted OMVs. These results also suggest that other OMV-packaged OMPs may be involved in roles such as stress response, cell wall and plasmid maintenance, and extracellular transport.

## Introduction

*Cronobacter* species are opportunistic pathogens which cause infections in all age groups; however, neonates are at a higher risk and remain the most susceptible age group for life-threatening, invasive disease (Lai, 2001; Jason, 2012; Yan et al., 2012; Tall et al., 2014; Patrick et al., 2014). The genus contains seven species: *Cronobacter sakazakii, Cronobacter malonaticus, Cronobacter turicensis, Cronobacter muytjensii, Cronobacter dublinensis, Cronobacter universalis*, and *Cronobacter condimenti* (Iversen et al., 2008; Joseph et al., 2012); however, only *C. sakazakii, C. malonaticus*, and *C. turicensis* cause the majority of infantile illness which usually presents clinically as necrotizing enterocolitis, bacteremia, and meningitis (Yan et al., 2012; Tall et al., 2014). Infections such as pneumonia, septicemia, catheter-associated, and urinary tract infections in adults have also been reported (Lai, 2001; Patrick et al., 2014). Additionally, *Cronobacter* species have been associated with other foods and food production environments, such as the manufacture of dried foods (e.g., dried milk, cheese powders, spices, and herbs) thereby posing a risk to susceptible consumers (Noriega et al., 1990; Himelright et al., 2002; El-Sharoud et al., 2008; Osaili et al., 2009; Yan et al., 2012).

Formation and secretion of outer membrane vesicles (OMVs) by Gram-negative bacteria are reported to occur under both *in vitro* and *in vivo* growth conditions (Kim et al., 2015). Outer membrane vesicles are thought to be involved in pathogenesis by delivering LPS components, outer membrane proteins (OMPs), effector molecules, such as toxins, enzymes, adhesins, and DNA/RNA to target host cells (Kim et al., 2015). Several studies have shown the relationship of OMV secretion to bacterial pathogenesis. The delivery of heat-labile enterotoxins, hemolysin (a RTX-like toxin) and cytolysin A expressed by *Escherichia coli*, and delivery of Cholera toxin to enterocytes are well-studied examples (Bladen and Waters, 1963; Bayer and Anderson, 1965; Chatterjee and Das, 1967; Knox et al., 1996; Kolling and Matthews, 1999; Chatterjee and Chaudhuri, 2011; Avila-Calderón et al., 2015; Kim et al., 2015). Two published reports describe the secretion of OMVs and their contribution to pathogenesis by three *C. sakazakii* strains (Alzahrani et al., 2015; Ye et al., 2015). However, not much is known about the molecular biology and prevalence of OMP genes among other pathogenic *Cronobacter* species including the three major pathogens, *C. sakazakii, C. malonaticus*, and *C. turicensis*. Singh et al. (2015) recently reviewed the role of two OMPs, OmpA and OmpX in adherence and invasion of epithelial and endothelial cells of human and animal origins. Accordingly, these OMPs play a role in both adherence and invasion through receptors that are located in both apical and basolateral host cell membrane surfaces. In fact, Schwechheimer and Kuehn (2015) suggest that packaging of the OMV contents is a more deliberate process than a stochastic event or that they are released just from cells due to lysis. They further delineate the process where it is conceivable (yet still hypothetical) that the production of OMVs by cells may results in different populations of OMVs with varying contents or “cargos.” None the less, there is now enough support to state that OMV production is a genuine bacterial secretion process.

In this report, we describe the expression of OMVs, identify proteins associated with OMVs isolated from *C. sakazakii, C. malonaticus*, and *C. turicensis*, and present results of PCR, sequence, and pan genomic microarray analyses of the OMP genes of 240 *Cronobacter* and phylogenetically-related strains. Our data support the working hypothesis that OMVs are novel secretion systems used by *Cronobacter* to deliver important components (e.g., LPS and proteins) involved in the host-pathogen interaction and may also play a role in stress response, cell wall and plasmid maintenance, and extracellular transport.

## Materials and methods

### Bacterial strains

The strains evaluated in this study consisted of 204 *C. sakazakii*, 12 *C. turicensis*, nine *C. malonaticus*, three *C. dublinensis*, and one strain each for *C. muytjensii, C. universalis*, and *C. condimenti*, which were obtained from the FDA laboratory culture collection. All these isolates were confirmed as *Cronobacter* species according to the proposed classification scheme as suggested by Iversen et al. (2008) and Joseph et al. (2012) using a Gram-negative card analyzed with the Vitek-2 Compact instrument (BioMerieux, Hazelwood, MO) and its version 5.03 software. Nine phylogenetically-related species representative of *Salmonella enterica* serovar Typhimurium, *Klebsiella pneumoniae, Citrobacter freundii, Siccibacter turicensis, Franconibacter helveticus* (two strains), and *Franconibacter pulveris* (two strains) were also included. All of the *Cronobacter* strains possessed the zinc metalloprotease (*zpx*) gene, a genus-specific target we previously reported (Kothary et al., 2007; Stephan et al., 2014). The species identity of the isolates was also confirmed using the species-specific *rpoB* PCR assays as described by Stoop et al. (2009) and Lehner et al. (2012) and the *cgcA* species-specific PCR assay as described by Carter et al. (2013). These 231 strains used in the study were isolated from clinical, food, or environmental sources and from diverse geographical locations and have been previously described by Franco et al., Yan et al., and Jarvis et al (Mullane et al., 2008; Franco et al., 2011; Jarvis et al., 2011, 2013; Yan et al., 2015a). The isolates were also subjected to RepF1B plasmidotyping (Franco et al., 2011) and molecular serogrouping (Mullane et al., 2008; Jarvis et al., 2011, 2013), the results of which further corroborated results of the *rpoB*- and *cgcA*- based PCR species identification assays.

### Bacterial culture maintenance

Frozen bacterial cultures were stored at −80°C in Trypticase soy broth (BBL, Becton Dickinson, Franklin Lakes, NJ) supplemented with 1% NaCl (TSBS) and 50% glycerol. For propagation, frozen cultures were rapidly thawed and sub-cultured onto plates containing Trypticase soy agar (TSA, BBL) supplemented with 1% NaCl (TSAS) or Luria Bertani (LB) agar (LBA, BBL), and the plates were incubated for 16–18 h at 37°C.

### OMV isolation

Using a modification of the procedure described by Kwon et al. (2009) for the isolation of OMVs from *Acinetobacter baumannii*, OMVs were obtained from *C. sakazakii* strain BAA-894, *C. malonaticus* strain LMG23826^T^ and *C. turicensis* strain LMG23827^T^ by culturing these bacterial strains on TSAS and incubating the plates at 37°C for 18 h. The stationary phase grown cells were harvested in saline using a sterile plastic spreader, and the OMVs were isolated by differential centrifugation. Briefly, the cells and cellular debris were removed by centrifugation (10,000 × g for 15 min at 4°C), and OMVs contained in the resultant supernatant were further concentrated by ultracentrifugation at 150,000 × g for 3 h at 4°C. The pellets were suspended in 500 μl 20 mM Tris-HCl buffer (pH 8.0) and qualitatively assessed by electron microscopy and SDS-PAGE. Aside for the occasional flagellum, the OMVs observed in these preparations were similar to those observed in the original whole cell preparations.

### Electron microscopy

Whole cell and the isolated OMV preparations were negatively stained and examined by electron microscopy. Whole cell suspensions were prepared in 0.5% sodium phosphotungstate (pH 6.8) and OMV suspensions were diluted 1:2 in 20 μl of a 1% (wt/vol) sodium phosphotungstate (pH 6.8) staining solution (final concentration was 0.5% sodium phosphotungstic acid). A single drop (containing approximately 15 μl) of the whole cell or OMV stained suspensions was then placed onto the surface of a 300 mesh, 0.25% Formvar-coated, carbon-coated copper grid (Electron Microscopy Sciences, Hatfield, PA). After drying for 1 min, excess fluid was removed from the grid surface with filter paper, and specimens were allowed to air dry before being examined in a JEOL 1011 transmission electron microscope (JEOL USA, Inc., Peabody, MA) operating at an accelerating voltage of 80 kV. Images were recorded using a Gatan model 785 side-mounted, ES1000W Erlangshen CCD camera using Digital Micrograph™ of the Gatan Microscopy Suite software version (Gatan, Inc., Pleasanton, CA).

### Sodium dodecyl sulfate (SDS)-polyacrylamide gel electrophoresis (PAGE) and western blotting

SDS-PAGE was performed on OMV preparations (containing approximately 2.5–5 mg/ml protein) using 8–25% gradient gels in the PhastSystem (GE Healthcare, Piscataway, NJ). Molecular weights of the reduced and denatured OMV proteins from the three *Cronobacter* spp. were estimated by using the relative mobility method (Weber et al., 1972). Western blotting of OMV protein samples separated by SDS-PAGE was carried out by electro-blotting onto a Problott (Applied Biosystems, Foster City, CA) membrane in a transfer buffer (10 mM of 3-[cyclohexylamino]-1-propane sulfonic acid containing 10% methanol, pH 11).

### N-terminal amino acid sequencing

The N-terminal amino acid sequences of the excised Coomassie blue-stained OMV protein bands from Western blots were determined by classical Edman degradation reactions using a Shimadzu PPSQ-33A Protein Sequencer (Columbia, MD). Computations for protein homology and identity analyses were performed using NCBI BLAST analysis.

### Bacterial genomic preparations used for PCR and DNA microarray analyses

Strains were grown overnight at 37°C in 5 ml of TSBS on an Innova shaker incubator (Eppendorf North America, Hauppauge, NY) at 160 rpm. Genomic DNA was purified from 2 ml of the culture using a robotic QIAcube workstation with its automated Qiagen DNeasy chemistry (Qiagen, Germantown, MD) following the manufacturer's recommendations. Typically, 5–15 μg of purified genomic DNA was recovered in a final elution volume of 200 μl. For PCR analysis, 1–2 μl of the genomic preparations were used as DNA templates. For microarray analysis, the purified DNA was further concentrated using an Amicon Ultracel-30 membrane filter (30,000 molecular weight cutoff, 0.5 ml, Millipore Corp. Billerica, MA) to a final volume of approximately 10–25 μl.

### PCR assay development for the presence of gene targets

PCR primers specifically targeting the *groEL, ompA, ompC, ompX, mipA* genes, the conjugative plasmid transfer protein gene, and an outermembrane (OM) autotransporter protein gene(s) were designed by using NCBI Primer-BLAST software (Ye et al., 2012). PCR primer names, sequences, amplicon sizes, and gene target names are shown in Table 1. The primers used for the PCR amplification were synthesized by Integrated DNA Technologies (Coralville, IA). All PCR reactions were carried out using the GoTaq® Green Master Mix (Promega Corp., Madison, WI), which consisted of a 25 μl reaction mix having 1 unit of GoTaq Hotstart DNA polymerase, 1.5 mM MgCl_2_, and 200 μM of each dNTP. Primers were added at 1 μM concentration each along with 1 μl of the genomic DNA (containing approximately 50 ng DNA/25 μl reaction) that served as a template. In all PCR reactions, the polymerase was activated by using a 3 min. incubation step at 94°C, followed by 25 cycles of denaturation at 94°C for 30 s and annealing and extension steps according to the PCR reaction parameters as described in Table 1. For each reaction, a final extension step of 5 min. at the cycle extension temperature was included.

**Table 1:** **PCR primers used in this study**.

**Primer gene target**	**Forward/Reverse primers**	**Sequence(5′-3′)**	**Amplicon size(bp)**	**Annealing/extension cycle parameters**
*ompA*	ompAFw2	TCAAAGCTCAGGGCGTACAG	542	58°C for 30s/72°C for 30s
	ompARv2	ACCCTGGTTGTAAGCGTCAG		
*ompC*	ES15ompCFw	CCGATACCGATAGCGCCGTA	282	58°C for 30s/72°C for 30s
	ES15ompCRv	GCGGACATCCATGACAGACA		
*ompX*	ompXFw1	CGTAGATGCTTGCCCAGTCA	288	58°C for 30s/72°C for 30s
	ompXRv1	CAGCACTGGCTTGTGTTCTG		
*groEL*	groELFw	GGCGGTACCGTTATCTCTGA	722	52.5°C for 30s/72°C for 1m
	groELRv	ATACCTGCAGCGCCTAAGTC		
*mipA*	mipAright1-Fw	CTCTATCGCTACACCAACGGC	305	62°C for 30s/72°C for 30s
	mipAright4-Rv	GTATAGGTCACGCCGGTAGA		
*ctp*	ctpLRightFw	CAGCACCCGTGTGGATCAAA	740	51°C for 30s/72°C for 70s
	ctpLRightRv	CCCAGGTTTCGTCTTCACCA		
*omatp*	omatpFwpp2	CCGTCGATACCCACATCCAG	537	60°C for 30s/72°C for 40s
	omatpRvFpp2	CAGCGAGAAATCGAAAGCGG		

### Microarray design

The microarray used in this study is an Affymetrix MyGeneChip Custom Array (Affymetrix design number:FDACRONOa520845F) and was developed utilizing the whole genome sequences and 18 plasmids of 15 *Cronobacter* strains as previously described (Tall et al., 2015; Yan et al., 2015b).

### Microarray hybridization

Genomic DNA was fragmented by incubating at 37°C for 1 min. in a 20 μl reaction containing 1 × One-Phor-All Plus Buffer (GE Healthcare) and 0.01 units DNase I (GE Healthcare) as previously described (Jackson et al., 2011), and further modified by Tall et al. (2015). The fragmented DNA was heat-inactivated at 99°C for 15 min, and was 3′-end labeled by adding 4 μl of 5 × terminal transferase buffer (Promega), 1 μl 1 mM biotin-11-ddATP (PerkinElmer NEL508), and 2 μl (60 units) of terminal transferase enzyme (Promega). The labeling was carried out for 4 h at 37°C followed by heat inactivation at 98°C for 1 min.

Hybridizations were performed according to the Affymetrix GeneChip Expression Analysis Technical Manual for a 49-format array (Affymetrix, 2014). Following hybridization, wash and stain procedures were carried out on an Affymetrix FS-450 fluidics station using the mini_prok2v1_450 fluidics script (Affymetrix, 2014). Reagents for washing and staining were prepared according to the GeneChip® Expression Analysis Technical Manual (Affymetrix, 2014). The following modifications were made to the wash and stain procedure: Streptavidin solution mix (vial 1) was replaced with SAPE solution mix (Life Technologies, Grand Island, NY). Arrays were scanned using Affymetrix GeneChip® Scanner 3000 running on AGCC software.

### Microarray data analysis

For each OMV gene represented on the microarray, probe set intensities were summarized using the Robust MultiArray Averaging (RMA) function in the Affymetrix package of R-Bioconductor as previously described (Bolstad et al., 2003). Briefly, RMA summarization of probe level data was carried out by performing three individual treatments on all of the experimental data (CEL file) in succession. Firstly, probe specific correction of the perfect matched (PM) probes was done using a model based on the observed intensities being the sum of signal and noise. Secondly, quantile normalization was performed on the corrected PM probe intensities. Finally, a median polishing algorithm was used to summarize the background-corrected, normalized probe intensities to generate a final probe set value.

### Accession numbers used to develop OMP gene PCR primers and *ompA* phylogenetic studies

The closed genomes of *C. sakazakii* ATCC BAA-894 (BioProject Accession PRJNA12720), *C. turicensis* z3032 (BioProject Accession PRJEA39965) and the draft genome for *C. malonaticus* LMG 23826 (BioProject Accession PRJNA89099) were used as references to develop PCR primers for the OMP gene PCR analysis. The accession numbers of the *Cronobacter* strains used to develop the phylogenetic analysis of *ompA* can be found in Supplemental Table 1.

## Results and discussion

During efforts to evaluate negatively-stained *Cronobacter* cell suspensions for the presence of fimbriae and other cell-surface associated adhesins by transmission electron microscopy (TEM), the existence of many extracellular vesicles, now known as OMVs, were observed cascading off the cell surfaces onto the sample support grid. These observations led us to use a combined N-terminal amino acid sequencing and genotyping approach to investigate the proteins and encoding genes associated with these bacterial surface structures in the three most prominent infantile *Cronobacter* pathogens: *C*. *sakazakii, C. malonaticus*, and *C. turicensis*.

### Electron microscopy

**Figure 1:**
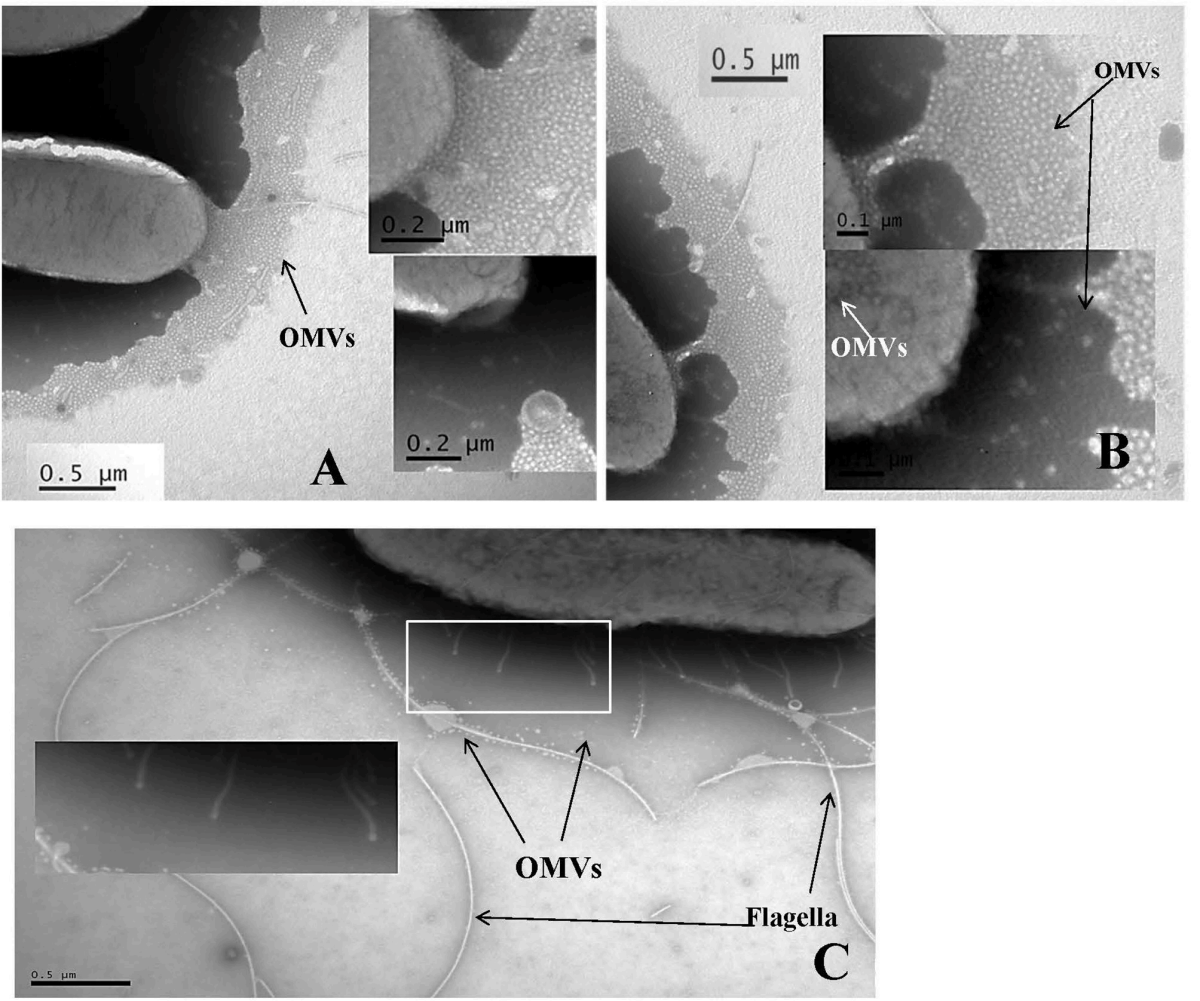
**Transmission electron micrograph of sodium phosphotungstate (pH 6.8) negatively stained *C. sakazakii* strain BAA-894 (A,B)** and *C. turicensis* strain LMG23827^T^ **(C)** cells showing outer membrane vesicles (labeled as OMVs) cascading off of the surface of the cells onto the support grid surface **(A,B)** or being pinched off of tendril like structures (identified by box and arrows in **C**). In **(A)**, Bar markers = 0.5 and 0.2 μm; in **(B)**, Bar markers = 0.5 and 0.2 μm; and in **(C)**, Bar marker = 0.5 μm.

**Figure 2:**
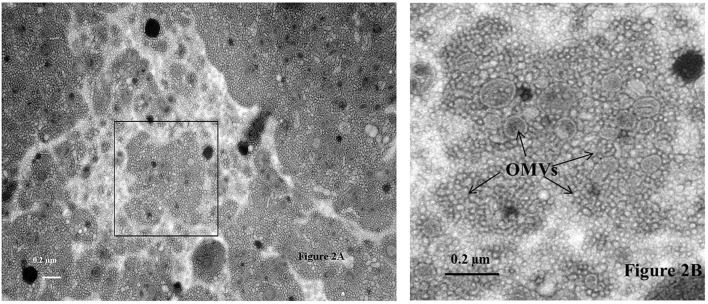
**Transmission electron micrograph of sodium phosphotungstate (pH 6.8) negatively stained outer membrane vesicles (Labeled as OMVs and identified by arrows) isolated from *C. sakazakii* strain BAA-894 using the protocol described in the text**. The area of the sample shown in the box within **(A)** is further magnified in **(B)**. Bar markers = 0.2 μm.

The generation of the first transmission electron photomicrograph of a bacterial cell is credited to Helmut Ruska, the brother of Nobel laureate Ernst Ruska (Knott and Genoud, 2013), who won the prize in 1986 for his work in electron optics, including the design of the first electron microscope. Though transmission electron microscopes were commercially available as early as 1939, it was not until the 1960s when researchers began to notice the presence of OMVs expressed by a variety of pathogenic Gram-negative bacteria including *Vibrio cholerae, E. coli*, and *Bacteroides fragilis* (Bladen and Waters, 1963; Bayer and Anderson, 1965; Chatterjee and Das, 1967; Knox et al., 1996; Kolling and Matthews, 1999; Chatterjee and Chaudhuri, 2011; Avila-Calderón et al., 2015; Kim et al., 2015). OMVs are thought to play several physiological and pathogenic functions in bacteria-bacteria and bacteria-host cell interactions however, as suggested by Schwechheimer and Kuehn they are not limited to just these roles (Schwechheimer and Kuehn, 2015). For example, Schwechheimer and Kuehn (2015) suggest that OMVs play functional roles in bacterial interactions with their environment(s), contributing to survival, nutrient acquisition, and ecological niche protection, including structural support in multispecies environments such as biofilms. TEM analysis of sodium phosphotungstate negatively-stained *Cronobacter* whole cell preparations showed an abundance of OMVs, and numerous OMVs were observed in these preparations, specifically as they cascaded off of each cell's surface onto the surface of the support grid (Figures 1A,B) or being pinched off of cell-associated membranous tendrils (Figure 1C). Occasionally, sheared and entrapped flagella were also seen in the OMV preparations. TEM evaluation of cells of all seven *Cronobacter* species produced similar OMVs (data not shown). Results of TEM analysis of partially isolated OMVs preparations from *C. sakazakii* strain BAA-894 (Figure 2) also demonstrates that the OMVs in these preparations were structurally similar to those observed in the negatively stained whole cell preparations. According to Mashburn-Warren and Whiteley, three main models for the expression and release of OMVs from the Gram-negative bacterial cell surface have been proposed (Mashburn-Warren and Whiteley, 2006). The first model posits that the OMVs originate from regions of the cell wall where there are no peptidoglycan-associated lipoproteins present (probably as a result of the outer membrane biosynthesis expanding faster than the underlying peptidoglycan layer). Then, these OMV-rich regions pinch off and are released from the cell surface. The second model proposes that the peptidoglycan fragments generated during normal cell wall turnover and/or repair are not efficiently transported back into the bacterial cytoplasm, thus leaving a region devoid of peptidoglycan. Turgor pressure resulting from a build-up of peptidoglycan within the periplasm would then cause blebbing of the outer membrane. The third model is thought to be specific to *Pseudomonas aeruginosa* where ionic interactions between *Pseudomonas* Quinolone Signal (PQS) and Mg^2+^ ions within the outer membrane enhances anionic repulsion between LPS molecules resulting in membrane blebbing (Mashburn-Warren et al., 2009). It was subsequently shown that PQS components are packaged within OMVs and these signaling components are thought to also be required for OMV formation (Mashburn-Warren et al., 2009). Though it is not known how OMVs are released from the *Cronobacter* cell surface, the vesicles observed in Figures 1, 2 could have been produced by mechanisms described in any of the proposed models. Schwechheimer and Kuehn (2015) have discussed in much greater detail the various models of OMV biogenesis, and various OMV structural components and their roles in the production of OMVs.

### SDS-PAGE analysis of OMVs

**Figure 3:**
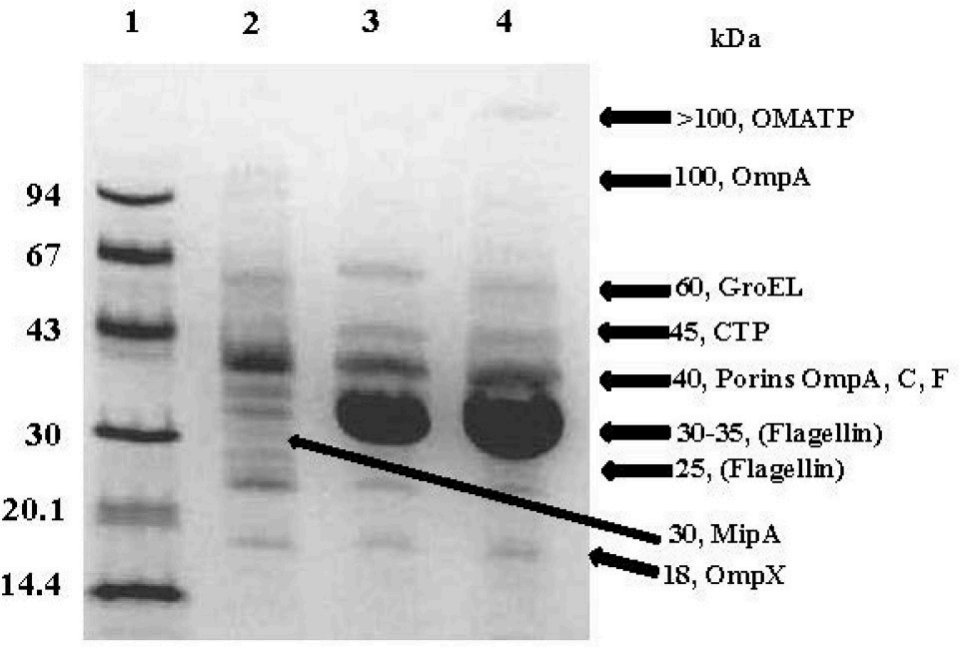
**Representative SDS-PAGE analysis of OMPs associated with OMVs isolated from *C. sakazakii* strain BAA-894, *C. turicensis* strain LMG23827^T^ and *C. malonaticus* strain LMG 23826^T^**. Lanes: 1, molecular mass markers (values at left are in kilodaltons); 2, *C. sakazakii* (4 μg); 3, *C. turicensis* (8 μg) and 3, *C. malonaticus* (5 μg). Molecular sizes (kDa) denoted on the right side of gel are indicative of the OMPs identified. Gel was stained with Coomassie brilliant blue R.

SDS-PAGE analysis of partially purified OMV preparations obtained from *C. sakazakii, C. malonaticus*, and *C. turicensis* (Figure 2) showed the presence of approximately 11 major protein bands, ranging in molecular weight (MW) from 18 to >100-kDa (Figure 3). The estimated MWs of these protein bands were 18, 25, 30, 30–35, 40 (three closely aligned protein bands), 50, 60, 100, and >100 kDa, and N-terminal amino acid sequencing and BLAST analysis (Table 2) identified these protein bands as OmpX, MipA, contaminating flagellin (Stephan et al., 2014, and 30–35 kDa), OmpA, OmpC, OmpE, OmpF, conjugative plasmid transfer protein (CTP), GroEL, an oligomer of OmpA, and an OM autotransporter protein (OMATP), respectively. The fact that known OMPs such as OmpA, C, E, F, and X were found associated with the isolated OMVs by SDS-PAGE and BLAST analysis strongly suggests that the isolation protocol used to obtain OMVs was successful in separating OMVs from cellular debris and other intracellular structures, except for the noted occasional entrapped flagella. Another interesting point can be made: many of the OMPs identified are OMPs which are related to the stress response. Schwechheimer and Kuehn address this in their review (Schwechheimer and Kuehn, 2015) where they suggest that OMV contents vary depending on the growth phase of the culture prior to OMV isolation. Because the cells used in the present study were more than likely at stationary phase of growth (overnight growth from a TSAS agar plate), OMVs shedding was maximized and their contents more reflective of cells growing under a stressful growth environment. This finding also explains why few cytoplasmic or cell membrane proteins were identified compared to OMPs reported in two previous studies reported by Alzahrani et al. (2015) and Ye et al. (2015). Though differences in protein abundance for *C. sakazakii* vs. that for *C. malonaticus* and *C. turicensis* were observed, collectively similar proteins were observed in the OMV preparations of each the three species. Other than the common flagellin and GroEL chaperonin proteins, the OMPs identified in the present study were not identified by Alzahrani et al. (2015). Quite possibly, the differences in types of OMV proteins could be ascribed to how the bacteria were grown in the two studies prior to isolation, i.e., stationary grown cells on TSAS used in this study vs. log phase grown cells in Brain Heart Infusion Broth or tryptic soy broth used by Alzahrani et al. and Ye et al., respectively (Alzahrani et al., 2015; Ye et al., 2015).

**Table 2:** **Summary of proteins associated with OMVs expressed by *Cronobacter* spp**.

**Molecular size (kDa)**	**BLASTp ID (GenBank Reference ID, aa protein length)**	**Sequence**	**% Identity^a^**	**Function**
18	OmpX (WP_004388453, 170 aa)	GTATVTGGYAQSDAQGV	100	Adhesin/Invasin
30	MipA (WP_054624653, 248 aa)	EGKLSVGAGAG	100	Peptidoglycan synthesis
40	OmpC, E, F^b^, 356 aa	AEIYNKLGXKTDLYGXVTAE	75–80	Porins
40/>100	OmpA (ANC61537, 377 aa)	APKDNTXYAGG	91	Adhesin/Invasin/Porin
50	CTP (WP_054624118, 426 aa)	ANTXAQAGNDA	82	Conjugative Plasmid- Transfer-Type IV SS
60	GroEL (WP_004387019, 547 aa)	AAKDVKFGNDA	100	Chaperonin
>100	OMATP (WP_069682366, 2,356 aa)	FTPDVTGSVQGQLVQ	80	Autotransporter secretion-Type V SS

a *% BLASTp Identity indicates the similarity of the sequenced N-terminal amino acid residues to those in data base*.

b *Reference sequences for these porin proteins are: ompF, CBA29682, 365 aa; ompE, ABU78340 and CBA28287, 357 and 361 aa; ompC, ABU78340, and WP_004385347, 357, and 356 aa, respectively*.

### OmpA, OmpC, OmpE, OmpF, and OmpX porin-like proteins

One of the three 40 kDa OMV protein bands and a 100 kDa protein band were identified as OmpA (Table 2). The first OMP described for *Cronobacter* was OmpA expressed by *E. sakazakii* strain 51329 (now *C. muytjensii*) by Mohan Nair and Venkitanarayanan (2006). BLAST analysis showed that each *Cronobacter* species possessed homologs of *ompA* (NCBI annotation identified the protein as encoding for an OmpA precursor protein). The 100 kDa protein possessed the same N-terminal amino acid sequence as the 40 kDa protein and these results suggest that the larger protein band represents an oligomer of the smaller protein band. Since the initial report by Mohan Nair and Venkitanarayanan (2006), *ompA* has also been shown to be a genus-specific PCR target and the protein shares homology with other porin-like OMPs common among the *Enterobacteriaceae*.

Similar to the OmpA, three additional proteins OmpC, OmpE, and OmpF also with MWs of 40 kDa were identified as porin-like proteins. Porin proteins span the outer membrane and act as a pore through which molecules can diffuse, and typically control the diffusion of small metabolites like sugars, ions, and amino acids, but some can also selectively transport a single group of molecules, or may be specific for one molecule (Klebba, 2005). For example, β-lactam and fluoroquinolone antibiotics must pass through porins to reach their targets in Gram-negative bacteria. Another OMP identified in the OMV preparations is OmpX with a MW of approximately 18-kDa. Vogt and Schulz (1999) described OmpX in *E. coli* as a member of a family of highly conserved bacterial proteins that promote bacterial adhesion to and entry into mammalian cells. Adherence factor functions have also been described for both OmpX and OmpA in *Cronobacter*. Recent reports (Mohan Nair and Venkitanarayanan, 2007; Kim and Loessner, 2008; Singamsetty et al., 2008; Kim et al., 2010) describing the invasion of *Cronobacter* into INT407 and Caco-2 cell lines have revealed that expression of OmpA, OmpX, and host actin filaments are required for invasion. In addition, *Cronobacter* invasion of HBMEC has been reported to require microtubules (Singamsetty et al., 2008). The findings reported here for OmpX and OmpA are similar to those reported by Ye et al. (2015).

### MltA

A 30 kDa protein was identified as a membrane-bound lytic transglycosylase, MltA-interacting protein, which belongs to the OmpV family of OMPs and is thought to be primarily involved in cell wall/outer membrane biosynthesis (Table 2). MltA-interacting protein is encoded by the gene *mipA* which is a member of a family of genes that encode for several bacterial MltA-interacting proteins. MltA-interacting proteins are responsible for the enlargement of the stress-bearing peptidoglycan layer of bacteria, and their activity depends on the coordinated interaction of murein synthases and hydrolases, as well as its interaction with the membrane-bound lytic transglycosylase, MltA. MltA-interacting proteins are also known to bind to outer membrane-associated penicillin binding proteins, such as PBP1B, a bifunctional murein transglycosylase/transpeptidase. Voller et al. (1999) propose that MltA-interacting proteins attach to PBP1B to form a complex and in *E. coli*, represent the first step in reconstitution of the hypothetical murein-synthesizing holoenzyme; this complex is thought to be responsible for the controlled growth of the peptidoglycan layer. The presence of this protein within OMVs further supports the association of the cell wall biosynthesis hypothesis proposed by Mashburn-Warren and Whiteley (2006), Mashburn-Warren et al. (2009).

### Conjugative plasmid transfer protein

A 50 kDa protein was identified as a conjugative plasmid transfer protein (CTP) (Table 2). Conjugative transfer of bacterial plasmids is the most effective way of horizontal gene transfer, and it is therefore considered to be one of the major causes for the increase in the multidrug antibiotic resistance, with homologies now being assigned to type IV secretion systems. The most noted conjugative transfer protein is the P-type conjugative transfer protein, TrbG which is associated with the *trb* locus of *Agrobacterium* Ti plasmids. The TrbG-like protein is a lipoprotein homolog of the F-type TraK protein (which is believed to be an outer membrane pore-forming secretin), as well as the *vir* system-encoded VirB9 protein. The protein is hypothesized to oligomerize to form a ring-like structure similar to other secretins, and several additional observations support the hypothesis that TraK interacts with TraV and TraB to form a complex in *E. coli* that spans the cell envelope from the outer membrane (TraV) through the periplasm (TraK) to the inner membrane (TraB) (Harris et al., 2001). Dorward et al. (1989) proposed that OMVs can also facilitate genetic exchange between bacteria. Indeed, DNA contained in OMVs has been successfully transferred into other bacterial cells, even between cells of different species (Kahn et al., 1983). As proposed by Olsen and Amano (2015), this represents a bacterial DNA delivery system that is not yet well recognized and documented. The presence of this protein within these OMVs complements the fact that the conjugative plasmids such as pESA2, pSP291-2, and pCTU2 are present in some *Cronobacter* spp. and in particular, the three strains analyzed in this study.

### GroEL

An approximately 60-kDa protein was identified as GroEL (Table 2). Skjærven et al. (2015) suggest that GroEL plays an essential role in the control of cellular stress and as a molecular chaperon, and is found in a large number of bacteria. In addition to its stress response function, GroEL is thought to be required for the proper folding of many proteins i.e., assist in refolding of heat/stress-damaged proteins (Zeilstra-Ryalls et al., 1991). In order to function properly, GroEL requires a second protein, the lid-like co-chaperon protein complex GroES. Speculatively, the association of GroEL with OMVs may aid in the proper folding of other OMPs, such as OmpX and OmpA, for delivery to host tissues. The genes *groES* (*mopA*) and *groEL* (*mopB*) of *E. coli* form an operon which was first defined by mutations affecting the morphogenesis of several bacteriophages. For example, both of the *groES/EL* gene products have been shown to be essential for bacteriophage head and tail assemblies (Fayet et al., 1989). Furthermore, Frisk et al. (1989) showed using whole-cell enzyme-linked immunosorbent assay and immnuoelectron microscopy that the expression of GroEL in *Haemophilus ducreyi* was associated with the cell surface. The finding of GroEL associated *Cronobacter* spp. OMVs support these results and possibly identifies a new cellular location in *Cronobacter* other than the cytoplasm as suggested by Alzahrani et al. (2015).

### OM autotransporter protein

Another large protein (>100 kDa) was identified as an OM autotransporter protein (OMATP). The secretion of proteins occurs in a number of different pathways in bacteria, and several secretion mechanisms have been ascribed to Gram-negative bacteria. The autotransporter secretion pathway (Lee and Schneewind, 2001) is a distinct secretion mechanism, in which an autotransporter protein moiety mediates the export of a protein through the outer membrane and the protein itself serves as the precursor of the secreted protein. Autotransporters have been implicated as important components/factors in mediating virulence mechanisms, such as the expression of adhesions used to colonize host cells, and actin-promoted bacterial mobility (Frisk et al., 1989). Autotransporters comprise three functional domains:**(1)** a N-terminal targeting domain (amino-terminal leader sequence) that functions as a signal peptide to mediate targeting to and translocation across the inner membrane; **(2)** a C-terminal translocation domain (carboxy-terminal) that forms a beta-barrel pore to allow for secretion (Henderson et al., 1998; Lee and Schneewind, 2001; Veiga et al., 2002); and **(3)** the secreted mature protein. The association of an autotransporter protein with the formation of OMVs supports the hypothesis that OMVs are another example of a bacterial secretion system. One of the most noted outer membrane protein autotransporter genes is *icsA* found on pWR100, the virulence plasmid of *Shigella* spp. This gene is responsible for the intracellular/intercellular bacterial movement by eliciting polar deposition of filamentous actin to bind to the bacterial cell surface in an actin-based motility (Bernardini et al., 1989). Thus, inside a host cell the nucleation and polymerization of actin molecules onto the *Shigella* cell surface by IcsA provides propulsion for intracellular movement and intercellular dissemination. However, little is known about the role and association of autotransporter proteins in *Cronobacter* pathogenesis.

### Distribution and prevalence of OMP genes by PCR

PCR analysis was used to demonstrate OMP encoding gene prevalence and distribution among 240 *Cronobacter* and phylogenetically-related strains. We observed that all of the *Cronobacter* species possessed genes for OMATP, OmpA, OmpC, and GroEL (Table 3). In contrast, 3 of 12 *C. dublinensis* strains; 2 of 16 *C. turicensis*; and 10 of the 19 phylogenetically-related strains were PCR-negative for *ompX*, while all other *Cronobacter* strains were PCR-positive for this target. Within the 19 phylogenetically related strains, isolates that were PCR-negative for gene targets include 18 for OMATP gene, 16 for *ompC*, 16 for *groEL*, 17 for *mipA*, and 19 for *ctp*, suggesting that the primers used to detect these gene targets mostly detected *Cronobacter*-specific gene regions. Overall, these results suggest that genes for these OMPs appear to be conserved among *Cronobacter* species; however conservation of *ompA*, and *ompX* may be extended to other members of the *Enterobacteriaceae*. These findings also support the growing body of literature which reports that OMP porin proteins such as OmpA, OmpC, OmpF, OmpX and the chaperonin GroEL are well conserved among members of this large family of Gram-negative bacteria, and also supports the findings reported by Alzahrani et al. (2015) and Ye et al. (2015).

**Table 3:** **Prevalence and distribution of OMP genes among 240 *Cronobacter* strains and phylogenetically-related species which were identified by PCR**.

**OMVs gene target**	**No. of isolates (%) per *Cronobacter* spp. and phylogenetically-related strains which possessed OMP vesicle PCR-positive alleles**								
	***C. mal* (%)**	***C. sak* (%)**	***C. tur* (%)**	***C. muy* (%)**	***C. con* (%)**	***C. dub* (%)**	***C. uni* (%)**	**Nearest neighbors (%)**	**Total No. positive (%)**
*omatp*	16 (100)	164 (100)	16 (100)	11 (100)	1 (100)	12 (100)	1 (100)	1 (5)	222 (93)
*ctp*	16 (100)	164 (100)	16 (100)	11 (100)	1 (100)	7 (58)	1 (100)	0 (0)	216 (90)
*groEL*	16 (100)	164 (100)	16 (100)	11 (100)	1 (100)	12 (100)	1 (100)	3 (16)	224 (93)
*mipA*	16 (100)	160 (98)	16 (100)	10 (91)	0 (0)	12 (100)	1 (100)	2 (11)	217 (90)
*ompA*	16 (100)	164 (100)	16 (100)	11 (100)	1 (100)	12 (100)	1 (100)	19 (100)	240 (100)
ompC	16 (100)	164 (100)	16 (100)	11 (100)	1 (100)	12 (100)	1 (100)	3 (16)	224 (93)
*ompX*	16 (100)	164 (100)	14 (88)	11 (100)	1 (100)	9 (75)	1 (100)	9 (47)	225 (94)
Total no. isolates	16	164	16	11	1	12	1	19	240

### Prevalence and distribution of OMP genes by DNA microarray analysis

**Figure 4:**
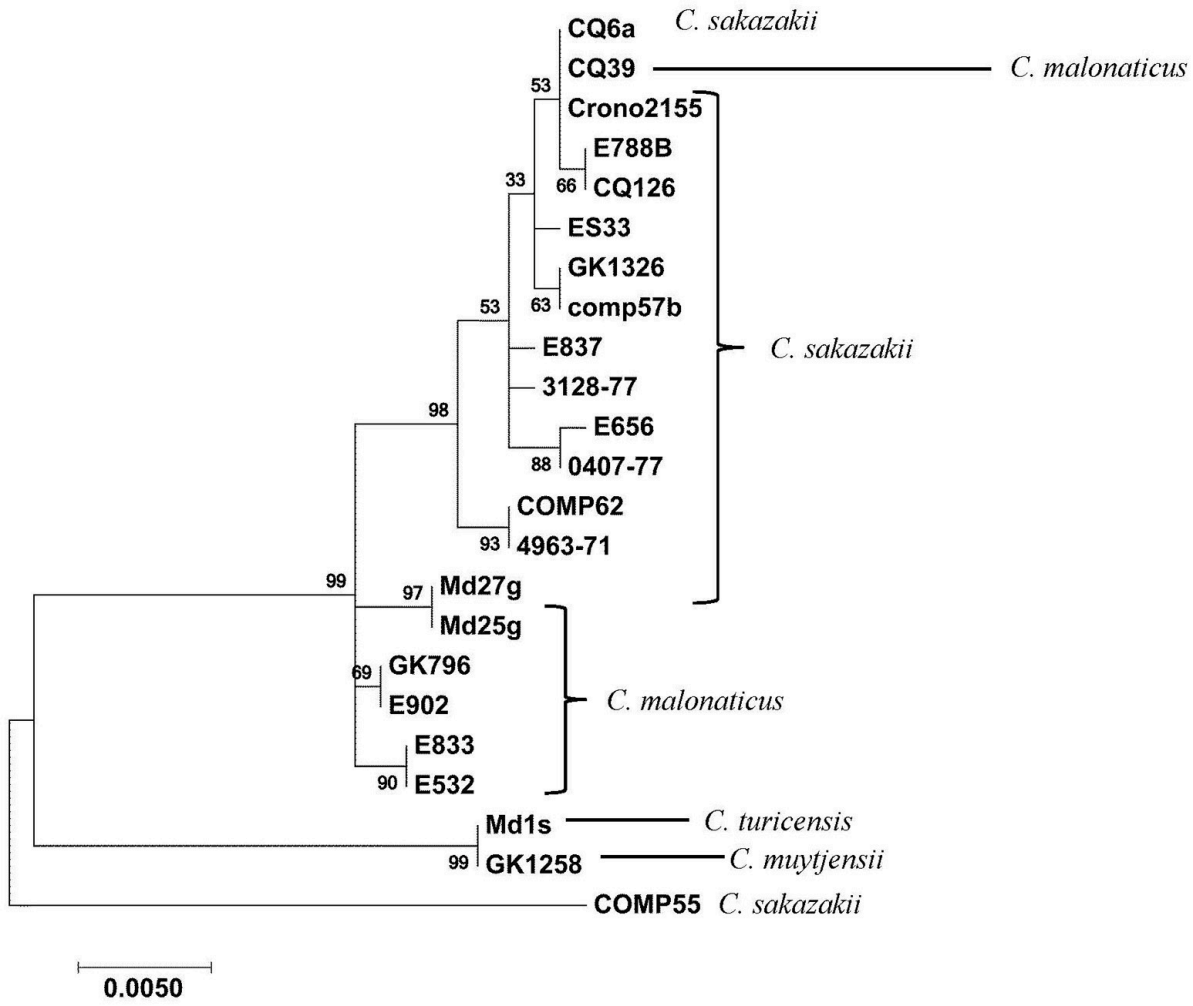
***ompA*-gene based clustering of *Cronobacter* isolates highlight intra- and inter-species sequence diversity**. Twenty-one alleles of *ompA* representing the 77 genomes analyzed in this work were aligned using ClustalW and clustered in the Maximum-likelihood method (Tamura and Nei, 1993) using the tools available on MEGA7 phylogenetic suite (Kumar et al., 2016). One thousand forty-four positions were evaluated for allelic information across the span of *ompA* gene sequences for the 21 strains. The bootstrap consensus (values located on the nodes) in the tree are inferred from 500 replicates. These values are taken to represent the evolutionary history of the taxa analyzed using the option available in MEGA7. The manual annotation of the tree highlights the intraspecies differences in *C. sakazakii, C. turicensis, C. muytjensii*, and *C. malonaticus*. The other members of the individual groups are listed in Supplemental Table 1 and those strains listed in the table and are identified by an asterisk (^**^) indicate those strains which had their *ompA* nucleotide sequences translated. Additionally those strains listed in this table, identified by a single (^*^), represent the strains included in this figure.

Recently, a novel pan genomic DNA microarray was developed to augment the development of next generation sequencing methods that can rapidly detect and characterize *Cronobacter* species and to be used as a highly discriminatory characterization and identification tool for the protection of public health in outbreaks or in source attribution investigations (Tall et al., 2015). Using this microarray, the presence of the OMP genes among 240 *Cronobacter* and phylogenetically-related species was determined. The microarray has 59 OMP alleles (probesets) representing all seven *Cronobacter* species. A summary of the hybridization results after the interrogation of these strains with 13 of these OMP gene probesets is shown in Table 4 and the presence/absence results demonstrate that particular alleles such as the OMATP gene from *C. sakazakii* and *ompC* and *ompF* from *C. malonaticus* captured on the microarray are more species-specific, while *groEL* and *ompA* alleles from *C. turicensis* and the *ompC* allele from *C. muytjensii* are highly conserved among all of the species. Nucleotide diversity of *ompA* sequences from 23 *Cronobacter* strains (representative of the 77 strains reported in this work) is shown in Figure 4 and different clusters of *Cronobacter* strains were observed. In most cases, there is a species-wise grouping of the *ompA* alleles as illustrated in the annotated tree. The diversity of *C. sakazakii* is demonstrated and emerging differences among some *C. malonaticus, C. turicensis, and C. muytjensii* strains were also noted. For example, the *ompA* allele of *C. malonaticus* strain CQ39 grouped with *C. sakazakii* strains instead of other *C. malonaticus* strains; and *C. turicensis* strain Md1s grouped with *C. muytjensii* strain GK1258. These two observations suggest that a wide nucleotide sequence divergence exists among *ompA* genes of various *Cronobacter* spp. This nucleotide sequence divergence was also captured by the microarray analysis and the results of the phylogenetic analysis using just the microarray OMP probesets is shown in Supplemental Figure 1. Interestingly, the *Cronobacter* and phylogenetically-related strains grouped as respective species into seven major clusters. For example *C. sakazakii, C. malonaticus, C. turicensis*, and *C. univiersalis* strains grouped into clusters 1–3, 4 and 6, 5, and 3, respectively. In contrast, *C. dublinensis, C. muytjensii*, and *C. condimenti* grouped as separate species clades within cluster 7. Lastly, the phylogenetically-related strains grouped as non-*Cronobacter* (labeled as nearest neighbors) species clades within clusters 1 and 7. This phylogenetic tree again agrees with similar phylogenetic analyses generated using whole genome sequence information (Grim et al., 2013; Stephan et al., 2014), and demonstrates the usefulness of the microarray to study the phylogenetic relationship of a specific set of alleles (Stephan et al., 2014; Tall et al., 2015; Yan et al., 2015b). In order to determine whether the various *ompA* genes translate into different proteins, the deduced 348 amino acid sequences of 10 *ompA* nucleotide sequences representing strains shown in Figure 4 were compared (Data not shown). Two interesting points can be made from this comparative sequence analysis: (1) A large cluster of 27 *C. sakazakii* strains of multiple serotypes (CsakO:2, CsakO:4, and ND), and sequence types (ST4, ST218, and ND) possessed an *ompA* gene similar in nucleotide and deduced protein sequence to that of Clonal Complex 4 *C. sakazakii* strains; and (2) several *Cronobacter* species possessed similar *ompA* genes; for example, *C. sakazakii* strain Md27g and *C. malonaticus* strain Md25g clustered together as did *C. turicensis* strain Md1s and *C. muytjensii* strains GK1258 and GK 1257. Comparing *ompA* gene sequences from subgroups of *C. sakazakii* and from *Cronobacter* species revealed nucleotide divergence at the intra- and inter-species levels. Any functional significance associated with these changes has to be elucidated further.

**Table 4:** **Microarray hybridization results of representative OMP alleles (probe sets) obtained from the interrogation of 240 *Cronobacter* and phylogenetically-related strains**.

**OMP**	***Cronobacter***	**NCBI Ref**.	**No. of isolates per *Cronobacter* spp. positive with specified OMP allele (%)**								
**Gene**	**Species**	**No**.	***C. mal***	***C. sak***	***C. tur***	***C. muy***	***C. con***	***C. dub***	***C. uni***	**Nearest neighbors**	**Total No. pos**.
*omatp*	*sakazakii*	ABU77334	0 (0)	204 (100)	1 (8)	0 (0)	0 (0)	0 (0)	0 (0)	0 (0)	205 (85)
*CTP*	*sakazakii*	ABU75399	7 (78)	204 (100)	6 (50)	1 (100)	0 (0)	3 (100)	1 (100)	9 (100)	231 (96)
*groEL*	*condimenti*	ABU75458	3 (33)	103 (50)	0 (0)	1 (100)	1 (100)	0 (0)	0 (0)	9 (100)	117 (49)
*groEL*	*turicensis*	ABU75458	9 (100)	204 (100)	12 (100)	1 (100)	0 (0)	3 (100)	1 (100)	8 (89)	238 (99)
*mipA*	*dub. Laus*.	ABU77421	0 (0)	0 (0)	0 (0)	0 (0)	0 (0)	3 (100)	0 (0)	0 (0)	3 (1)
*mipA*	*condimenti*	ABU77421	0 (0)	0 (0)	0 (0)	0 (0)	1 (100)	0 (0)	0 (0)	0 (0)	1 (0.4)
*ompA*	*dub. Laus*.	ABU76166	1 (11)	1 (0.5)	0 (0)	0 (0)	0 (0)	3 (100)	1 (100)	7 (78)	13 (5)
*ompA*	*turicensis*	ABU79362	9 (100)	204 (100)	12 (100)	1 (100)	0 (0)	3 (100)	1 (100)	0 (0)	239 (99)
*ompC*	*muytjensii*	ABU76243	9 (100)	204 (100)	12 (100)	1 (100)	1 (100)	2 (67)	1 (100)	9 (100)	239 (99)
*ompC*	*malonaticus*	ABU76243	7 (78)	0 (0)	0 (0)	0 (0)	0 (0)	0 (0)	0 (0)	0 (0)	7 (3)
*ompF*	*turicensis*	ABU77659	0 (0)	0 (0)	2 (17)	0 (0)	0 (0)	0 (0)	0 (0)	0 (0)	2 (1)
*ompF*	*malonaticus*	ABU77659	1 (11)	0 (0)	0 (0)	0 (0)	0 (0)	0 (0)	0 (0)	0 (0)	1 (0.4)
*ompX*	*sakazakii*	ABU77741	5 (56)	204 (100)	0 (0)	0 (0)	0 (0)	0 (0)	0 (0)	0 (0)	209 (87)
Total no. isolates			9	204	12	1	1	3	1	9	240

In summary, OMVs are released from both pathogenic and non-pathogenic Gram-negative bacterial cells by possibly several, yet unclear, mechanisms. In *Cronobacter* species, these vesicles are thought to be formed when the OM protrudes, and sometimes encapsulates both periplasmic and outer membrane bound components within their structures. Early on, these structures were thought to be artifacts but currently, OMVs are recognized as structures possessing very diverse functions. Foremost, OMVs can be regarded as a secretion system used by bacteria to communicate with host cells and other bacterial cells. Though the secretion of lipids, OMPs, and signaling molecules is achieved, the actual delivery system with these components is quite unique in that their contents are protected inside the lumen of the OMVs from the environment, and the intact OMVs can then be delivered directly to specific host cell receptors as suggested by Alzahrani et al. (2015). Many organisms use OMVs to secrete virulence factors. As such, bacteria may actively regulate their OMV contents to influence the host-pathogen interaction(s). For example, once *Cronobacter* gains entry into the systemic circulation either through the breakdown of the intestinal barrier *via* necrotizing enterocolitis or active adherence, invasion and translocation of enterocytes as suggested by Giri et al. (2012), OMVs may be released in abundant numbers and result in the delivery of OmpA and OmpX to target host cell receptors, such as the endothelial cells of the capillary bed making up the blood-brain-barrier (BBB). If there is an overwhelming abundant number of OMVs with associated components such as LPS and OMPs which are then presented to the host's immune system, a pro-inflammatory response may be stimulated which could lead to a fatal “cytokine storm.” Such immune responses have been previously described (Townsend S. M. et al., 2007; Mittal et al., 2009) and were shown to be inefficient in fighting intracellular infections. In order to determine whether LPS was associated with the OMVs, a preliminary experiment (data not shown) using a Pyrosate test kit for the detection of LPS (Associates of Cape Cod, Inc., MA) showed that LPS was present in the OMVs preparations. Speculatively, if a pro-inflammatory “cytokine storm” did occur (as suggested here by these preliminary results that LPS was associated with OMVs, and by evidence previously reported, Townsend S. M. et al., 2007; Mittal et al., 2009; Alzahrani et al., 2015) at the surface of endothelial cells within the capillary beds of the BBB in the presence of *Cronobacter* cells expressing OmpA and OmpX, alteration in the physiology and permeability of these cells could occur and lead to translocation (Giri et al., 2012) and bacterial invasion of the meninges. In addition, the infection may be further exacerbated by the interaction of the host's immune cells and *Cronobacter*, for example, the pathogen's survival and persistence within the phagocytic cells may further contibute to meningitis (Townsend S. M. et al., 2007; Mittal et al., 2009). The existence of *Cronobacter* and LPS components in PIF manufacturing environments (Townsend S. et al., 2007) is an excellent example of this intersection where man-made processes have selected for an organism's colonization and persisitence within this environment and the subsequent contamination of PIF products which are utlimately consumed by humans. It is this dynamic juncture where this bacterium, within its man-made environment, and its comtamination of a food product may contribute precisely to what King (2013) describes as the “triple threat” facing the overall health of humans as illustrated in the One Health Initiative. The global nature of our food supply further raises public health concerns about the risk of transporting microbes; i.e., microbes can move globally faster than their disease incubation periods, and the dangers to both human and animal health is increasing, with both food and water as potential vehicles for the dissemination of pathogens. Finally, the One Health approach is also proactive, preventive, and compliments nicely with the guidelines described in the Food Safety Modernization act of 2011 which will help to shift food safety attentions “upstream” to the ecological, animal, and environmental sources responsible for foodborne illnesses and, therefore, helps us to identify the most effective points for the initiation of food safety actions.

## Author contributions

All authors (MK, GG, JG, IP, PR, LH, QY, HC, BL, EP, YY, TC, LC, VS, SF, and BT) contributed to the drafting of the manuscript. MK, GG, JG, IP, PR, LH, QY, HC, BL, EP, YY, TC, LC, SBF, FN, and VS carried out the data acquisition, analysis and interpretation of data. HC, BL, EP, YY, TC, LC, performed PCR and Microarray experiments and JG, IP, and GG interpreted the results. HC, BL, EP, YY, TC, and MK performed SDS-PAGE and protein sequencing experiments. HC, BL, EP, YY, TC, and BT performed the whole genome sequencing experiments and GG interpreted the results. All authors contributed to the study concept and design; and critical revision of the manuscript for important intellectual content.

### Conflict of interest statement

The authors declare that the research was conducted in the absence of any commercial or financial relationships that could be construed as a potential conflict of interest.

## References

[B1] Affymetrix (2014). Expression Analysis Technical Manual with Specific Protocols for use with the Hybridization Wash and Stain Kit. Available online at: http://media.affymetrix.com/support/downloads/manuals/expression_analysis_technical_manual.pdf

[B2] Alzahrani H. Winter J. Boocock D. De Girolamo L. Forsythe S. J. (2015). Characterization of outer membrane vesicles from a neonatal meningitic strain of *Cronobacter sakazakii*. FEMS Microbiol. Lett. 362:fnv085 10.1093/femsle/fnv085 26023200

[B3] Avila-Calderón E. D. Araiza-Villanueva M. G. Cancino-Diaz J. C. Lopez-Villegas E. O. Sriranganathan N. Boyle S. M.. (2015). Roles of bacterial membrane vesicles. Arch. Microbiol. 197, 1–10. 10.1007/s00203-014-1042-7 25294190

[B4] Bayer M. E. Anderson T. F. (1965). The surface structure of *Escherichia coli*. Proc. Natl. Acad. Sci. U.S.A. 54, 1592–1599. 10.1073/pnas.54.6.1592 5326381 PMC300519

[B5] Bernardini M. L. Mounier J. d'Hauteville H. Coquis-Rondon M. Sansonetti P. J. (1989). Identification of icsA, a plasmid locus of *Shigella flexneri* that governs bacterial intra- and intercellular spread through interaction with F-actin. Proc. Natl. Acad. Sci. U.S.A. 86, 3867–3871. 10.1073/pnas.86.10.3867 2542950 PMC287242

[B6] Bladen H. A. Waters J. F. (1963). Electron microscopic study of some strains of *Bacteroides*. J. Bacteriol. 86, 1339–1344. 14086111 10.1128/jb.86.6.1339-1344.1963 PMC283651

[B7] Bolstad B. M. Irizarry R. A. Astrand M. Speed T. P. (2003). A comparison of normalization methods for high density oligonucleotide array data based on variance and bias. Bioinformatics 19, 185–193. 10.1093/bioinformatics/19.2.185 12538238

[B8] Carter L. Lindsey L. A. Grim C. J. Sathyamoorthy V. Jarvis K. G. Gopinath G.. (2013). Multiplex PCR assay targeting a diguanylate cyclase-encoding gene, *cgcA*, to differentiate species within the genus *Cronobacter*. Appl. Environ. Microbiol. 79, 734–737. 10.1128/AEM.02898-12 23144142 PMC3553758

[B9] Chatterjee D. Chaudhuri K. (2011). Association of cholera toxin with *Vibrio cholerae* outer membrane vesicles which are internalized by human intestinal epithelial cells. FEBS Lett. 585, 1357–1362. 10.1016/j.febslet.2011.04.017 21510946

[B10] Chatterjee S. N. Das J. (1967). Electron microscopic observations on the excretion of cell-wall material by *Vibrio cholerae*. J. Gen. Microbiol. 49, 1–11. 10.1099/00221287-49-1-1 4168882

[B11] Dorward D. W. Garon C. F. Judd R. C. (1989). Export and intercellular transfer of DNA via membrane blebs of *Neisseria gonorrhoeae. J Bacteriol*. 171, 2499–2505 (1989). Erratum in: J. Bacteriol. 171, 4104 10.1128/jb.171.5.2499-2505.1989 PMC209926 2496108

[B12] El-Sharoud W. M. El-Din M. Z. Ziada D. M. Ahmed S. F. Klena J. D. (2008). Surveillance and genotyping of *Enterobacter sakazakii* suggest its potential transmission from milk powder into imitation recombined soft cheese. J. Appl. Microbiol. 105, 559–566. 10.1111/j.1365-2672.2008.03777.x 18312564

[B13] Fayet T. Ziegelhoffer T. Georgopoulos C. (1989). The groES and groEL heat shock gene products of *Escherichia coli* are essential for bacterial growth at all temperatures. J. Bacteriol. 171, 1379–1385. 10.1128/jb.171.3.1379-1385.1989 2563997 PMC209756

[B14] Franco A. A. Hu L. Grim C. J. Gopinath G. Sathyamoorthy V. Jarvis K. G.. (2011). Characterization of putative virulence genes on the related RepFIB plasmids harbored by *Cronobacter* spp. Appl. Environ. Microbiol. 77, 3255–3267. 10.1128/AEM.03023-10 21421789 PMC3126477

[B15] Frisk A. Ison C. A. Lagergård T. (1989). GroEL Heat Shock Protein of *Haemophilus ducreyi*: association with cell surface and capacity to bind to Eukaryotic Cells. Infect. Immun. 66, 1252–1257. 9488422 10.1128/iai.66.3.1252-1257.1998 PMC108042

[B16] Giri C. P. Shima K. Tall B. D. Curtis S. Sathyamoorthy V. Hanisch B.. (2012). *Cronobacter* spp. (previously *Enterobacter sakazakii*) invade and translocate across both cultured human intestinal epithelial cells and human brain microvascular endothelial cells. Microb. Pathog. 52, 140–147. 10.1016/j.micpath.2011.10.003 22023990

[B17] Grim C. J. Kotewicz M. L. Power K. A. Gopinath G. Franco A. A. Jarvis K. G.. (2013). Pan-genome analysis of the emerging foodborne pathogen *Cronobacter* spp. suggests a species-level bidirectional divergence driven by niche adaptation. BMC Genomics. 14:366. 10.1186/1471-2164-14-366 23724777 PMC3680222

[B18] Harris R. L. Hombs V. Silverman P. M. (2001). Evidence that F-plasmid proteins TraV, TraK and TraB assemble into an envelope-spanning structure in *Escherichia coli*. Mol. Microbiol. 42, 757–766. 10.1046/j.1365-2958.2001.02667.x 11722740

[B19] Henderson I. R. Navarro-Garcia F. Nataro J. P. (1998). The great escape: structure and function of the autotransporter proteins. Trends Microbiol. 6, 370–378. 10.1016/S0966-842X(98)01318-3 9778731

[B20] Himelright I. Harris E. Lorch V. V. Anderson M. Jones T. Craig A.. (2002). *Enterobacter sakazakii* infections associated with the use of powdered infant formula—Tennessee, 2001. Morb. Mortal. Wkly Rep. 51, 297–300. 12002167

[B21] Iversen C. Mullane N. McCardell B. Tall B. D. Lehner A. Fanning S. (2008). *Cronobacter* gen. nov., a new genus to accommodate the biogroups of *Enterobacter sakazakii*, and proposal of *Cronobacter sakazakii* gen. nov., comb. nov., *Cronobacter malonaticus* sp. nov., *Cronobacter turicensis* sp. nov., *Cronobacter muytjensii* sp. nov., *Cronobacter dublinensis* sp. nov., Cronobacter genomospecies 1, and of three subspecies, *Cronobacter dublinensis* ssp. dublinensis ssp. nov., *Cronobacter dublinensis* ssp. lausannensis ssp. nov. and *Cronobacter dublinensis* ssp. lactaridi ssp. nov. Intern. J. Syst. Evol. Microbiol. 58, 1442–1447. 10.1099/ijs.0.65577-0 18523192

[B22] Jackson S. A. Patel I. R. Barnaba T. LeClerc J. E. Cebula T. A. (2011). Investigating the global diversity of *Escherichia coli* using a multi-genome DNA microarray platform with novel gene prediction strategies. BMC Genomics 12:349. 10.1186/1471-2164-12-349 21733163 PMC3146454

[B23] Jarvis K. G. Grim C. J. Franco A. A. Hu L. Gopinath G. Sathyamoorthy V.. (2011). Molecular characterization of *Cronobacter lipopolysaccharide* O-antigen gene clusters and development of serotype-specific PCR assays. Appl. Environ. Microbiol. 77, 4017–4026. 10.1128/AEM.00162-11 21531829 PMC3131661

[B24] Jarvis K. G. Yan Q. Q. Grim C. J. Power K. A. Franco A. A. Hu L.. (2013). Identification and characterization of five new molecular serogroups of *Cronobacter* spp. Foodborne Path Dis. 10, 343–352. 10.1089/fpd.2012.1344 23566272

[B25] Jason J. (2012). Prevention of invasive Cronobacter infections in young infants fed powdered infant formulas. Pediatrics 130, e1076–e1084. 10.1542/peds.2011-3855 23045556

[B26] Joseph S. Cetinkaya E. Drahovska H. Levican A. Figueras M. J. Forsythe S. J. (2012). *Cronobacter condimenti* sp. nov., isolated from spiced meat, and *Cronobacter universalis* sp. nov., a species designation for *Cronobacter* sp. genomospecies 1, recovered from a leg infection, water and food ingredients. Intern. J. Syst. Evol. Microbiol. 62, 1277–1283. 10.1099/ijs.0.032292-0 22661070

[B27] Kahn M. E. Barany F. Smith H. O. (1983). Transformasomes: specialized membranous structures that protect DNA during *Haemophilus* transformation. Proc. Natl. Acad. Sci. U.S.A. 80, 6927–6931. 10.1073/pnas.80.22.6927 6316334 PMC390099

[B28] Kim J. H. Lee J. Park J. Gho Y. S. (2015). Gram-negative and Gram-positive bacterial extracellular vesicles. Semin. Cell Dev. Biol. 40, 97–105. 10.1016/j.semcdb.2015.02.006 25704309

[B29] Kim K. Kim K.-P. Choi J. Lim J.-A. Lee J. Hwang S.. (2010). Outer membrane proteins (OmpA) and X (OmpX) are essential for basolateral invasion of *Cronobacter sakazakii*. Appl. Environ. Microbiol. 76, 5188–5198. 10.1128/AEM.02498-09 20543055 PMC2916488

[B30] Kim K. P. Loessner M. J. (2008). *Enterobacter sakazakii* invasion in human intestinal Caco-2 cells requires the host cytoskeleton and is enhanced by disruption of tight junction. Infect. Immun. 76, 562–570. 10.1128/IAI.00937-07 18070906 PMC2223463

[B31] King L. J. (2013). Combating the triple threat: the need for a One Health approach. Microbiol. Spectr. 1:OH-0012-2012. 10.1128/microbiolspec.OH-0012-2012 26184817

[B32] Klebba P. E. (2005). The porinologist. J. Bacteriol. 187, 8232–8236. 10.1128/JB.187.24.8232-8236.2005 16321927 PMC1317029

[B33] Knott G. Genoud C. (2013). Is EM dead? J. Cell Sci. 126, 4545–4552. 24124192 10.1242/jcs.124123

[B34] Knox K. W. Vesk M. Work E. (1996). Relation between excreted lipolysaccharide complexes and surface structures of a lysine-limited culture of *Escherichia coli*. J. Bacteriol. 92, 1206–1217. 10.1242/jcs.124123 PMC276396 4959044

[B35] Kolling G. L. Matthews K. R. (1999). Export of virulence genes and Shiga toxin by membrane vesicles of *Escherichia coli* O157:H7. Appl. Environ. Microbiol. 65, 1843–1848. 10223967 10.1128/aem.65.5.1843-1848.1999 PMC91264

[B36] Kothary M. H. McCardell B. A. Frazar C. D. Deer D. Tall B. D. (2007). Characterization of the zinc-containing metalloprotease Encoded by *zpx* and development of a species-specific detection method for *Enterobacter sakazakii*. Appl. Environ. Microbiol. 73, 4142–4151. 10.1128/AEM.02729-06 17483271 PMC1932767

[B37] Kumar S. Stecher G. Tamura K. (2016). MEGA7: molecular evolutionary genetics analysis version 7.0 for bigger datasets. Mol. Biol. Evol. 33, 1870–1874. 10.1093/molbev/msw054 27004904 PMC8210823

[B38] Kwon S.-O. Gho Y. Lee J. Kim S. (2009). Proteome analysis of outer membrane vesicles from a clinical *Acinetobacter baumannii* isolate. FEMS Microbiol. Lett. 297, 150–156. 10.1111/j.1574-6968.2009.01669.x 19548894

[B39] Lai K. K. (2001). *Enterobacter sakazakii* infections among neonates, infants, children, and adults. Case reports and a review of the literature. Med. (Baltimore) 80, 113–122. 10.1097/00005792-200103000-00004 11307587

[B40] Lee V. T. Schneewind O. (2001). Protein secretion and the pathogenesis of bacterial infections. Genes Develop. 15, 1725–1752. 10.1101/gad.896801 11459823

[B41] Lehner A. Fricker-Feer C. Stephan R. (2012). Identification of the recently described *Cronobacter condimenti* by a rpoB based PCR system. J. Med. Microbiol. 61, 1034–1035. 10.1099/jmm.0.042903-0 22466029

[B42] Mashburn-Warren L. M. Howe J. Brandenburg K. Whiteley M. (2009). Structural requirements of the *Pseudomonas* quinolone signal for membrane vesicle stimulation. J. Bacteriol. 191, 3411–3414. 10.1128/JB.00052-09 19286801 PMC2687154

[B43] Mashburn-Warren L. M. Whiteley M. (2006). Special delivery: vesicle trafficking in prokaryotes. Mol. Microbiol. 61, 839–846. 10.1111/j.1365-2958.2006.05272.x 16879642

[B44] Mittal R. Bulgheresi S. Emami C. Prasadarao N. V. (2009). *Enterobacter sakazakii* targets DC-SIGN to induce immunosuppressive responses in dendritic cells by modulating MAPKs. J. Immunol. 183, 6588–6599. 10.4049/jimmunol.0902029 19846880 PMC2796599

[B45] Mohan Nair M. K. Venkitanarayanan K. S. (2006). Cloning and sequencing of the ompA gene of *Enterobacter sakazakii* and development of an ompA-targeted PCR for rapid detection of *Enterobacter sakazakii* in infant formula. Appl. Environ. Microbiol. 72, 2539–2546. 10.1128/AEM.72.4.2539-2546.2006 16597955 PMC1449048

[B46] Mohan Nair M. K. Venkitanarayanan K. S. (2007). Role of OmpA and host cytoskeleton in the invasion of human intestinal epithelial cells by *Enterobacter sakazakii*. Pediatr. Res. 62, 664–669. 10.1203/PDR.0b013e3181587864 17957161

[B47] Mullane N. O'Gaora P. Nally J. E. Iversen C. Whyte P Wall, P. G.. (2008). Molecular analysis of the *Enterobacter sakazakii* O-antigen gene locus. Appl. Environ. Microbiol. 74, 3783–3794. 10.1128/AEM.02302-07 18441119 PMC2446543

[B48] Noriega F. R. Kotloff K. L. Martin M. A. Schwalbe R. S. (1990). Nosocomial bacteremia caused by *Enterobacter sakazakii* and *Leuconostoc mesenteroides* resulting from extrinsic contamination of infant formula. Ped. Infect Dis. J. 9, 447−449. 2114609

[B49] Olsen I. Amano A. (2015). Outer membrane vesicles-offensive weapons or good Samaritans? J. Oral Microbiol. 7:27468. 10.3402/jom.v7.27468 25840612 PMC4385126

[B50] Osaili T. M. Shaker R. R. Al-Haddaq M. S. Al-Nabulsi A. A. Holley R. A. (2009). Heat resistance of *Cronobacter* species (*Enterobacter sakazakii*) in milk and special feeding formula. J. Appl. Microbiol. 107, 928–935. 10.1111/j.1365-2672.2009.04271.x 19320941

[B51] Patrick M. E. Mahon B. E. Greene S. A. Rounds J. Cronquist A. Wymore K.. (2014). Incidence of *Cronobacter* spp. infections, United States, 2003-2009. Emerg. Infect. Dis. 20, 1520–1523. 10.3201/eid2009.140545 25148394 PMC4178417

[B52] Schwechheimer C. Kuehn M. J. (2015). Outer-membrane vesicles from Gram-negative bacteria: biogenesis and functions. Nat. Rev. Microbiol. 10, 605–619. 10.1038/nrmicro3525 PMC5308417 26373371

[B53] Singamsetty W. Y. Shimada H. Prasadarao N. V. (2008). Outer membrane protein A expression in *Enterobacter sakazakii* is required to induce microtubule condensation in human brain microvascular endothelial cells for invasion. Microbiol. Path. 45, 181–191. 10.1016/j.micpath.2008.05.006 18606523 PMC2536595

[B54] Singh N. Goel G. Raghav M. (2015). Insights into virulence factors determining the pathogenicity of *Cronobacter sakazakii*. Virulence 6, 433–440. 10.1080/21505594.2015.1036217 25950947 PMC4601314

[B55] Skjærven L. Cuellar J. Martinez A. Valpuesta J. M. (2015). Dynamics, flexibility, and allostery in molecular chaperonins. FEBS Lett. 589, 2522–2532. 10.1016/j.febslet.2015.06.019 26140986

[B56] Stephan R. Grim C. J. Gopinath G. R. Mammel M. K. Sathyamoorthy V. Trach L. H. (2014). Re-examination of the taxonomic status of *Enterobacter helveticus* sp. nov., *Enterobacter pulveris* sp. nov., and *Enterobacter turicensis* sp. nov. as members of Cronobacter: proposal of two new genera *Siccibacter* gen. nov. and *Franconibacter* gen. nov. and descriptions of *Siccibacter turicensis* sp. nov., *Franconibacter helveticus* sp. nov., and *Franconibacter pulveris* sp. nov. Intern. J. Syst. Evol. Microbiol. 64, 3402–3410. 10.1099/ijs.0.059832-0 PMC4179279 25028159

[B57] Stoop B. Lehner A. Iversen C. Fanning S. Stephan R. (2009). Development and evaluation of *rpoB* based PCR systems to differentiate the six proposed species within the genus *Cronobacter*. Intern. J. Food Microbiol. 136, 165–168. 10.1016/j.ijfoodmicro.2009.04.023 19467725

[B58] Tall B. D. Chen Y. Yan Q. Q. Gopinath G. R. Grim C. J. Jarvis K. G.. (2014). *Cronobacter*: an emergent pathogen causing meningitis to neonates through their feeds. Sci. Prog. 97, 154–172. 10.3184/003685014X13994743930498 25108996 PMC10365370

[B59] Tall B. D. Gangiredla J. Gopinath G. R. Yan Q. Chase H. R. Lee B. (2015). Development of a custom-designed, pan genomic DNA microarray to characterize strain-level diversity among *Cronobacter* spp. Front. Ped. 3:36 10.3389/fped.2015.00036 PMC4415424 25984509

[B60] Tamura K. Nei M. (1993). Estimation of the number of nucleotide substitutions in the control region of mitochondrial DNA in humans and chimpanzees. Mol. Biol. Evol. 10, 512–526. 8336541 10.1093/oxfordjournals.molbev.a040023

[B61] Townsend S. Caubilla Barron J. Loc-Carrillo C. Forsythe S. (2007). The presence of endotoxin in powdered infant formula milk and the influence of endotoxin and *Enterobacter sakazakii* on bacterial translocation in the infant rat. Food Microbiol. 24, 67–74. 10.1016/j.fm.2006.03.009 16943096

[B62] Townsend S. M. Hurrell E. Gonzalez-Gomez I. Lowe J. Frye J. G. Forsythe S.. (2007). *Enterobacter sakazakii* invades brain capillary endothelial cells, persists in human macrophages influencing cytokine secretion and induces severe brain pathology in the neonatal rat. Microbiology 153, 3538–3547. 10.1099/mic.0.2007/009316-0 17906151

[B63] Veiga E. Sugawara E. Nikaido H. de Lorenzo V. Fernandez L. A. (2002). Export of autotransported proteins proceeds through an oligomeric ring shaped by C-terminal domains. EMBO J. 21, 2122–2131. 10.1093/emboj/21.9.2122 11980709 PMC125980

[B64] Vogt J. Schulz G. E. (1999). The structure of the outer membrane protein OmpX from *Escherichia coli* reveals possible mechanisms of virulence. Structure 7, 1301–1309. 10.1016/S0969-2126(00)80063-5 10545325

[B65] Voller W. von Rechenberger M. Holtje J. V. (1999). Demonstration of molecular interactions between the murein polymerase PBP1B, the lytic transglycosylase MltA, and the scaffolding protein MipA of *Escherichia coli*. J. Biol. Chem. 274, 6726–6734. 10.1074/jbc.274.10.6726 10037771

[B66] Weber K. Pringle J. R. Osborn M. (1972). Measurement of molecular weights by electrophoresis on SDS-acrylamide gel. Meth. Enzymol. 26, 3–27. 10.1016/S0076-6879(72)26003-7 4680711

[B67] Yan Q. Q. Jarvis K. G. Chase H. R. Hébert K. Trach L. H. Lee C. (2015a). A proposed harmonized LPS molecular-based serotyping scheme for *Cronobacter* to aid in tracking its presence in manufacturing facilities and our food supply, and support clinical findings. Food Microbiol. 50, 38–43. 10.1016/j.fm.2015.03.003 25998813

[B68] Yan Q. Q. Condell O. Power K. Butler F. Tall B. D. Fanning S. (2012). *Cronobacter* species (formerly known as *Enterobacter sakazakii*) in powdered infant formula: a review of our current understanding of the biology of this bacterium. J. Appl. Microbiol. 113, 1–15. 10.1111/j.1365-2672.2012.05281.x 22420458

[B69] Yan Q. Q. Wang J. Gangiredla J. Cao Y. Martins M. Gopinath G. R. (2015b). Comparative genotypic and phenotypic analysis of *Cronobacter* species cultured from four powdered infant formula production facilities: indication of patho-adaptation along the food chain. Appl. Environ. Microbiol. 81, 4388–402. 10.1128/AEM.00359-15 25911470 PMC4475896

[B70] Ye J. Coulouris G. Zaretskaya I. Cutcutache I. Rozen S. Madden T. L. (2012). Primer-BLAST: a tool to design target-specific primers for polymerase chain reaction. BMC Bioinformatics 13:134. 10.1186/1471-2105-13-134 22708584 PMC3412702

[B71] Ye Y. W. Gao J. Jiao R. Li H. Wu Q. Zhang J.. (2015). The Membrane proteins involved in virulence of *Cronobacter sakazakii* virulent G362 and attenuated L3101 isolates. Front. Microbiol. 6:1238. 10.3389/fmicb.2015.01238 26617581 PMC4637405

[B72] Zeilstra-Ryalls J. Fayet O. Georgopoulos C. (1991). The universally conserved GroE (Hsp60) chaperonins. Annu. Rev. Microbiol. 45, 301–325. 10.1146/annurev.mi.45.100191.001505 1683763

