# Supplementary material for: Analysis and Characterization of Proteins Associated with Outer Membrane Vesicles Secreted by Cronobacter spp
Source: Front Microbiol. 2017 Feb 9;8:134. doi: 10.3389/fmicb.2017.00134 (PMC5299011; doi:10.3389/fmicb.2017.00134)
Supplement: Supplementary file 1 [file Table1.pdf]

Supplemental Table 1. Strain names, CFSAN isolate names, strain species identities[a], NCBI Accession numbers, and NCBI Bioproject numbers used to develop the phylogenetic tree in Figure 4.

**Strain**	**GenBank Locus**	**NCBI Protein ID**	**NCBI BioSample ID**	**NCBI Isolate ID**	***Cronobacter* spp.**	**Serotype[b]**
MOD1-0407-77*	KU354351	ANC61565	SAMN04329627	CFSAN043075	sakazakii	O:1
MOD1-1121-73**	KU354405	ANC61619	SAMN04329632	CFSAN043080	sakazakii	O:2
MOD1-1241	KU354406	ANC61620	SAMN04329588	CFSAN043036	sakazakii	O:4
MOD1-1242**	KU354389	ANC61603	SAMN04329597	CFSAN043045	sakazakii	O:4
MOD1-206	KU354383	ANC61597	SAMN04329598	CFSAN043046	sakazakii	O:2
MOD1-207N	KU354404	ANC61618	SAMN04329599	CFSAN043047	sakazakii	O:2
MOD1-208N	KU354371	ANC61585	SAMN04329600	CFSAN043048	sakazakii	O:2
MOD1-2156	KU354369	ANC61583	SAMN04329601	CFSAN043049	sakazakii	O:3
MOD1-2157	KU354374	ANC61588	SAMN04329602	CFSAN043050	sakazakii	O:2
MOD1-228N	KU354386	ANC61600	SAMN04329603	CFSAN043051	sakazakii	O:2
MOD1-229N	KU354416	ANC61630	SAMN04329604	CFSAN043052	sakazakii	O:2
MOD1-254N	KU354410	ANC61624	SAMN04329589	CFSAN043037	sakazakii	O:1
MOD1-255N	KU354367	ANC61581	SAMN04329605	CFSAN043053	sakazakii	O:2
MOD1-302	KU354372	ANC61586	SAMN04329606	CFSAN043054	sakazakii	O:2
MOD1-303N	KU354396	ANC61610	SAMN04329607	CFSAN043055	sakazakii	O:2
MOD1-304N	KU354413	ANC61627	SAMN04329608	CFSAN043056	sakazakii	O:2
MOD1-305**	KU354403	ANC61617	SAMN04329590	CFSAN043038	sakazakii	O:1
MOD1-306N	KU354394	ANC61608	SAMN04329609	CFSAN043057	sakazakii	O:2
MOD1-3128-77*	KU354360	ANC61574	SAMN04329648	CFSAN043096	sakazakii	O:1
MOD1-4963-71*	KU354390	ANC61604	SAMN04329591	CFSAN043039	sakazakii	O:1
MOD1-607-A	KU354352	ANC61566	SAMN04329646	CFSAN043094	sakazakii	O:2

MOD1-C120505	KU354359	ANC61573	SAMN04329626	CFSAN043074	sakazakii	O:2
MOD1-CDC2006-06-25**	KU354373	ANC61587	SAMN04329595	CFSAN043043	sakazakii	O:2
MOD-1CDC2011-21-03	KU354345	ANC61559	SAMN04329596	CFSAN043044	sakazakii	O:2
MOD1-comp11	KU354364	ANC61578	SAMN04329633	CFSAN043081	sakazakii	O:2
MOD1-comp19	KU354395	ANC61609	SAMN04329634	CFSAN043082	sakazakii	O:2
MOD1-comp20	KU354349	ANC61563	SAMN04329635	CFSAN043083	sakazakii	O:2
MOD1-comp45b	KU354377	ANC61591	SAMN04329640	CFSAN043088	sakazakii	O:2
MOD1-comp46	KU354411	ANC61625	SAMN04329636	CFSAN043084	sakazakii	O:2
MOD1-comp49b	KU354354	ANC61568	SAMN04329641	CFSAN043089	sakazakii	O:2
MOD1-comp53b	KU354368	ANC61582	SAMN04329642	CFSAN043090	sakazakii	O:2
MOD1-comp54a	KU354370	ANC61584	SAMN04329643	CFSAN043091	sakazakii	O:2
MOD1-comp55*, **	KU354323	ANC61537	SAMN04329652	CFSAN043100	sakazakii	O:2
MOD1-comp57b*	KU354417	ANC61631	SAMN04329644	CFSAN043092	sakazakii	O:2
MOD1-comp59a	KU354402	ANC61616	SAMN04329645	CFSAN043093	sakazakii	O:2
MOD1-comp62*	KU354382	ANC61596	SAMN04329592	CFSAN043040	sakazakii	O:2
MOD1-CQ111	KU354375	ANC61589	SAMN04329610	CFSAN043058	sakazakii	O:3
MOD1-CQ126*	KU354391	ANC61605	SAMN04329611	CFSAN043059	sakazakii	O:3
MOD1-CQ127	KU354401	ANC61615	SAMN04329612	CFSAN043060	sakazakii	O:3
MOD1-CQ128	KU354418	ANC61632	SAMN04329613	CFSAN043061	sakazakii	O:3
MOD1-CQ39*, **	KU354407	ANC61621	SAMN04329651	CFSAN043099	malonaticus	O:1
MOD1-CQ6*	KU354392	ANC61606	SAMN04329614	CFSAN043062	sakazakii	O:2
MOD1-CQ6a	KU354414	ANC61628	SAMN04329615	CFSAN043063	sakazakii	O:2
MOD1-Crono-2148	KU354366	ANC61580	SAMN04329616	CFSAN043064	sakazakii	O:2
MOD1-Crono-2150	KU354347	ANC61561	SAMN04329617	CFSAN043065	sakazakii	O:2

MOD1-Crono-2152	KU354358	ANC61572	SAMN04329618	CFSAN043066	sakazakii	O:2
MOD1-Crono-2154	KU354346	ANC61560	SAMN04329619	CFSAN043067	sakazakii	O:2
MOD1-Crono-2155*	KU354365	ANC61579	SAMN04329620	CFSAN043068	sakazakii	O:2
MOD1-E532*	KU354327	ANC61541	SAMN04329580	CFSAN043028	malonaticus	O:1
MOD1-E654	KU354408	ANC61622	SAMN04329593	CFSAN043041	sakazakii	O:1
MOD1-E656*	KU354350	ANC61564	SAMN04329628	CFSAN043076	sakazakii	O:1
MOD1-E657	KU354412	ANC61626	SAMN04329594	CFSAN043042	sakazakii	O:1
MOD1-E755	KU354381	ANC61595	SAMN04329629	CFSAN043077	sakazakii	O:4
MOD1-E756	KU354380	ANC61594	SAMN04329630	CFSAN043078	sakazakii	O:4
MOD1-E758B[**]	KU354387	ANC61601	SAMN04329631	CFSAN043079	sakazakii	O:4
MOD1-E760A	KU354357	ANC61571	SAMN04329647	CFSAN043095	sakazakii	O:2
MOD1-E788B[*, **]	KU354348	ANC61562	SAMN04329621	CFSAN043069	sakazakii	O:2
MOD1-E831	KU354338	ANC61552	SAMN04329581	CFSAN043029	malonaticus	O:1
MOD1-E833*	KU354316	ANC61530	SAMN04329582	CFSAN043030	malonaticus	O:1
MOD1-E837[*, **]	KU354388	ANC61602	SAMN04329649	CFSAN043097	sakazakii	O:2
MOD1-E893	KU354385	ANC61599	SAMN04329622	CFSAN043070	sakazakii	O:2
MOD1-E902*	KU354276	ANC61490	SAMN04329587	CFSAN043035	malonaticus	O:2
MOD1-ES30	KU354376	ANC61590	SAMN04329623	CFSAN043071	sakazakii	O:2
MOD1-ES32	KU354355	ANC61569	SAMN04329624	CFSAN043072	sakazakii	O:2
MOD1-ES33[*, **]	KU354397	ANC61611	SAMN04329650	CFSAN043098	sakazakii	O:7
MOD1-ES34	KU354379	ANC61593	SAMN04329625	CFSAN043073	sakazakii	O:2
MOD1-GK1025B	KU354398	ANC61612	SAMN04329637	CFSAN043085	sakazakii	O:2
MOD1-GK1026	KU354353	ANC61567	SAMN04329638	CFSAN043086	sakazakii	O:2
MOD1-GK1257	KU354400	ANC61614	SAMN04329578	CFSAN043026	muytjensii	ND

MOD1-GK1258*	KU354272	ANC61486	SAMN04329579	CFSAN043027	muytjensii	ND
MOD1-GK1326*	KU354300	ANC61514	SAMN04329639	CFSAN043087	sakazakii	O:2
MOD1-GK1355	KU354313	ANC61527	SAMN04329583	CFSAN043031	malonaticus	O:5
MOD1-GK796*	KU354337	ANC61551	SAMN04329584	CFSAN043032	malonaticus	O:5
MOD1-GK962	KU354311	ANC61525	SAMN04329585	CFSAN043033	malonaticus	O:5
MOD1-Md1s*	KU354270	ANC61484	SAMN04329654	CFSAN043102	turicensis,	ND
MOD1-Md25g*	KU354278	ANC61492	SAMN04329586	CFSAN043034	malonaticus	O:2
MOD1-Md27g*	KU354281	ANC61495	SAMN04329653	CFSAN043101	sakazakii	ND

[a]Specific PCR assays as described by Stoop et al. (2009), Lehner et al. (2012) and Carter et al. (2013) were used to confirm the species identities of the isolates.

[b]Serotype was determined as described by Yan et al. (2015a)

* Strains denoted with an asterisk were used to develop the phylogenetic tree shown in Figure 4.

** Strains denoted with an a double asterisk were used to compare the amino acid seqeunces.

Stoop B, Lehner A, Iversen C, Fanning S, Stephan R. 2009. Development and evaluation of rpoB based PCR systems to differentiate the six proposed species within the genus *Cronobacter* . Intern J Food Microbiol 136:165-168.

Lehner A, Fricker-Feer C, Stephan R. 2012. Identification of the recently described Cronobacter condimenti by a rpoB based PCR system. J Med Microbiol 61:1034-1035. doi: 10.1099/jmm.0.042903-0. Epub 2012.

Carter L, Lindsey LA, Grim CJ, Sathyamoorthy V, Jarvis KG, Gopinath G, Lee C, Sadowski JA, Trach L, Pava-Ripoll M, McCardell BA, Tall BD, Hu L. 2013. Multiplex PCR assay targeting a diguanylate cyclase-encoding gene, cgcA, to differentiate species within the genus Cronobacter. Appl Environ Microbiol 79:734-737. doi: 10.1128/AEM.02898-12. Epub 2012 Nov 9.

Yan, Q. Q., Jarvis, K. G., Chase, H. R., & Hébert, K., et al. (2015a). A proposed harmonized LPS molecular-based serotyping scheme for *Cronobacter* to aid in tracking its presence in manufacturing facilities and our food supply, and support clinical findings.

Food Microbiol. 50, 38-43. doi: 10.1016/j.fm.2015.03.003. Epub 2015 Mar 17.
