# Supplementary material for: Analysis and Characterization of Proteins Associated with Outer Membrane Vesicles Secreted by Cronobacter spp
Source: Front Microbiol. 2017 Feb 9;8:134. doi: 10.3389/fmicb.2017.00134 (PMC5299011; doi:10.3389/fmicb.2017.00134)
Supplement: Supplementary file 2 [file Image1.PDF]

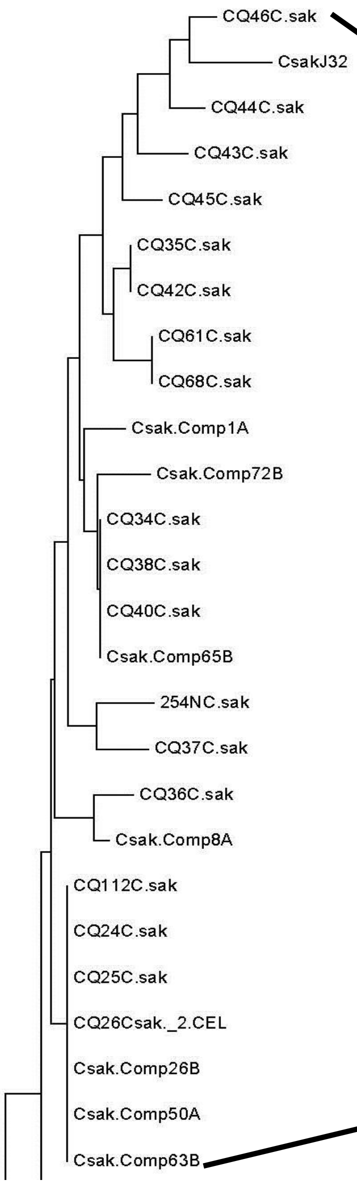

Strain	Species	Source	Serotype	ST
CQ46	*sakazakii*	PIF Facility, Environmental	CsakO:1	1
J32	*sakazakii*	Food, Baby food	CsakO:3	ND
CQ44	*sakazakii*	PIF Facility, Environmental	CsakO:1	1
CQ43	*sakazakii*	PIF Facility, Environmental	CsakO:1	1
CQ45	*sakazakii*	PIF Facility, Environmental	CsakO:1	1
CQ35	*sakazakii*	PIF Facility, Environmental	CsakO:1	1
CQ42	*sakazakii*	PIF Facility, Environmental	CsakO:1	1
CQ61	*sakazakii*	PIF Facility, Environmental	CsakO:1	1
CQ68	*sakazakii*	PIF Facility, Environmental	CsakO:1	ND
Comp1A	*sakazakii*	Dairy Facility, Environmental	CsakO:1	ND
Comp72B	*sakazakii*	Dairy Facility, Environmental	CsakO:2	ND
CQ34	*sakazakii*	PIF Facility, Environmental	CsakO:1	1
CQ38	*sakazakii*	PIF Facility, Environmental	CsakO:1	1
CQ40	*sakazakii*	PIF Facility, Environmental	CsakO:1	1
Comp65B	*sakazakii*	Dairy Facility, Environmental	CsakO:1	ND
254N	*sakazakii*	Clinical	CsakO:1	1
CQ37	*sakazakii*	PIF Facility, Environmental	CsakO:3	1
CQ36	*sakazakii*	PIF Facility, Environmental	CsakO:1	1
Comp38A	*sakazakii*	Dairy Facility, Environmental	CsakO:4	ND
CQ112	*sakazakii*	PIF Facility, Environmental	CsakO:4	1
CQ24	*sakazakii*	PIF Facility, Environmental	CsakO:4	1
CQ25	*sakazakii*	PIF Facility, Environmental	CsakO:4	1
CQ26	*sakazakii*	PIF Facility, Environmental	CsakO:4	1
Comp26B	*sakazakii*	Dairy Facility, Environmental	CsakO:1	ND
Comp50A	*sakazakii*	Dairy Facility, Environmental	CsakO:1	ND
Comp63B	*sakazakii*	Dairy Facility, Environmental	CsakO:1	ND

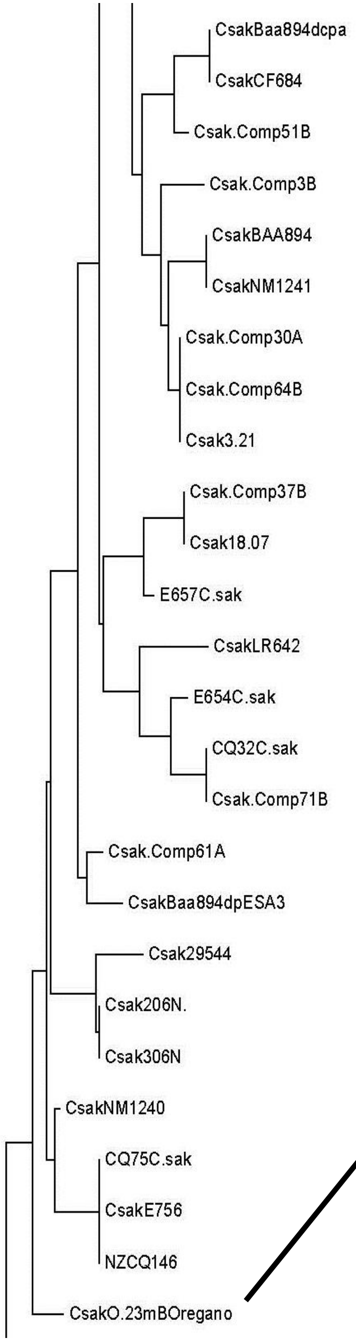

Strain	Species	Source	Serotype	ST
Baa894dcpa	*sakazakii*	Clinical, Δcpa	CsakO:1	
CF684	*sakazakii*	Chicken Feed	CsakO:1	ND
Comp51B	*sakazakii*	Dairy Facility, Environmental	CsakO:1	ND
Comp3B	*sakazakii*	Dairy Facility, Environmental	CsakO:1	ND
BAA894	*sakazakii*	Food, PIF	CsakO:1	1
NM1241	*sakazakii*	Clinical, Blood, Postmorten	CsakO:4	1
Comp30A	*sakazakii*	Dairy Facility, Environmental	CsakO:1	ND
Comp64B	*sakazakii*	Dairy Facility, Environmental	CsakO:1	ND
3.21	*sakazakii*	Food, Nuts	CsakO:1	ND
Comp37B	*sakazakii*	Dairy Facility, Environmental	CsakO:1	ND
18.07	*sakazakii*	Clinical, stool	CsakO:1	8
E657	*sakazakii*	Clinical	CsakO:1	1
LR642	*sakazakii*	Food, Sodium casinate	CsakO:3	ND
E654	*sakazakii*	Clinical	CsakO:1	1
CQ32	*sakazakii*	PIF Facility, Environmental	CsakO:1	1
Comp71B	*sakazakii*	Dairy Facility, Environmental	CsakO:1	ND
Comp61A	*sakazakii*	Dairy Facility, Environmental	CsakO:1	ND
Baa894dpESA3	*sakazakii*	Plasmid pESA3 cured derivative	CsakO:1	1
29544	*sakazakii*	Clinical, Child,Throat swab	CsakO:1	8
206N	*sakazakii*	Clinical	CsakO:2	4
306N	*sakazakii*	Clinical	CsakO:2	4
NM1240	*sakazakii*	Clinical, CSF	CsakO:4	4
CQ75	*sakazakii*	PIF Facility, Environmental	CsakO:3	Un-identified
E756	*sakazakii*	Clinical	CsakO:4	8
NZCQ146	*sakazakii*	PIF Facility, Environmental	ND	ND
O.23mBOregano	*sakazakii*	Oregano	CsakO:1	ND

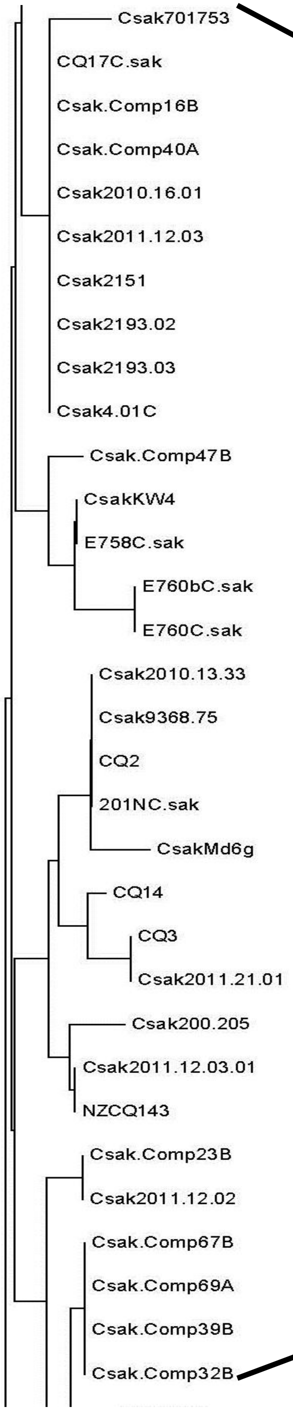

Strain	Species	Source	Serotype	ST
701753	*sakazakii*	PIF Facility, Environmental	CsakO:2	31
CQ17	*sakazakii*	PIF Facility, Environmental	CsakO:2	4
Comp16B	*sakazakii*	Dairy Facility, Environmental	CsakO:2	ND
Comp40A	*sakazakii*	Dairy Facility, Environmental	CsakO:2	ND
2010.16.01	*sakazakii*	Clinical, Brain exudate	CsakO:2	4
2011.12.03	*sakazakii*	Clinical, Rectal swab, Stool	CsakO:2	4
2151	*sakazakii*	Clinical, CSF	CsakO:2	4
2193.02	*sakazakii*	Clinical, Sputum	CsakO:2	4
2193.03	*sakazakii*	Clinical, CSF	CsakO:2	4
4.01C	*sakazakii*	Food, PIF	CsakO:2	218
Comp47B	*sakazakii*	Dairy Facility, Environmental	CsakO:7	ND
KW4	*sakazakii*	Food, Dried Seaweed	ND	ND
E758	*sakazakii*	Clinical	CsakO:4	8
E760b	*sakazakii*	Clinical	CsakO:2	264
E760	*sakazakii*	Clinical	CsakO:2	264
2010.13.33	*sakazakii*	Clinical,Blood	CsakO:2	4
9368.75	*sakazakii*	Unknown	CsakO:2	ND
CQ2	*sakazakii*	PIF Facility, Environmental	CsakO:2	4
201N	*sakazakii*	Clinical	CsakO:2	4
Md6g	*sakazakii*	Fly, *Musca domestica*, gut	CsakO:3	4
CQ14	*sakazakii*	PIF Facility, Environmental	CsakO:2	4
CQ3	*sakazakii*	PIF Facility, Environmental	CsakO:2	4
2011.21.01	*sakazakii*	Clinical, CSF	CsakO:2	4
200.205	*sakazakii*	Clinical, CSF	CsakO:2	4
2011.12.03.01	*sakazakii*	Clinical, CSF	CsakO:2	4
NZCQ143	*sakazakii*	PIF Facility, Environmental	ND	ND
Comp23B	*sakazakii*	Dairy Facility, Environmental	CsakO:2	ND
2011.12.02	*sakazakii*	Clinical, CSF	CsakO:2	4
Comp67B	*sakazakii*	Dairy Facility, Environmental	CsakO:4	ND
Comp69A	*sakazakii*	Dairy Facility, Environmental	CsakO:2	ND
Comp39B	*sakazakii*	Dairy Facility, Environmental	CsakO:2	ND
Comp32B	*sakazakii*	Dairy Facility, Environmental	CsakO:2	ND

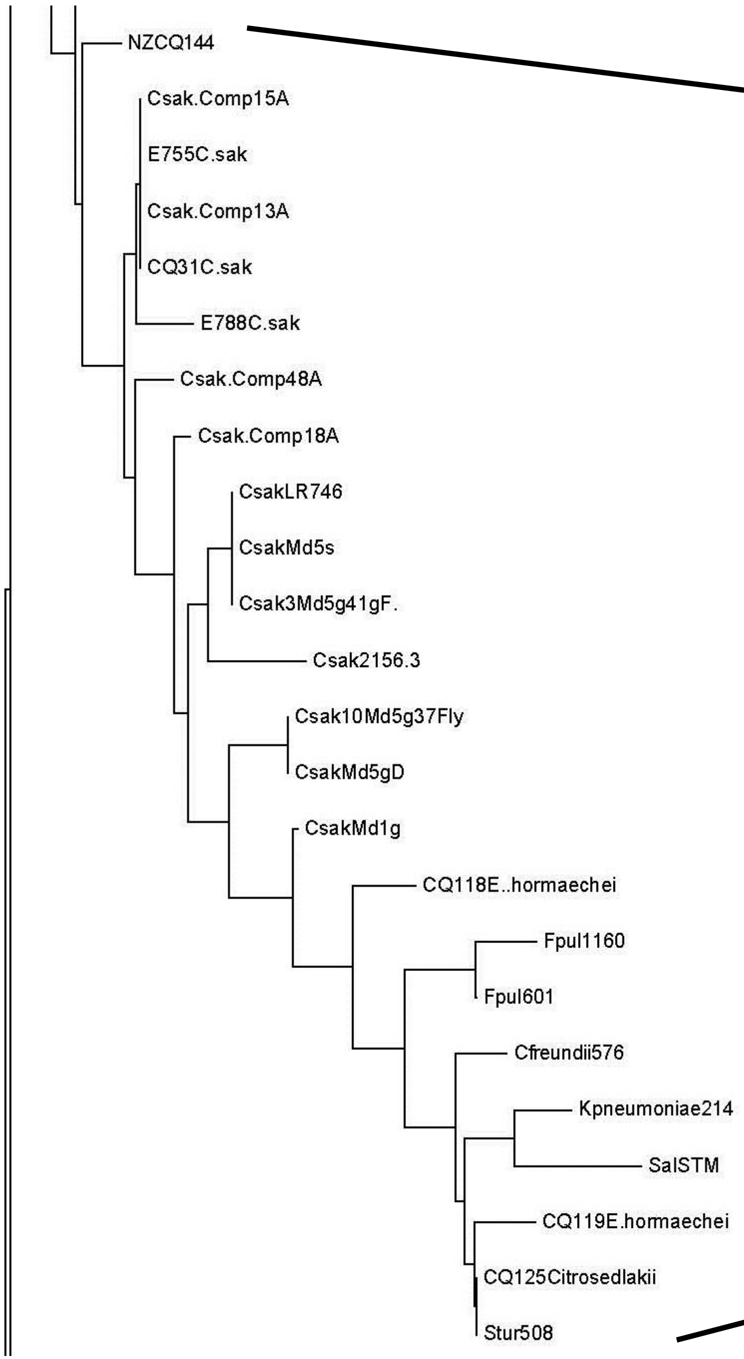

Strain	Species	Source	Serotype	ST
NZCQ144	*sakazakii*	PIF Facility, Environmental	ND	13
Comp15A	*sakazakii*	Environmental	CsakO:7	ND
E755	*sakazakii*	Clinical	CsakO:4	8
Comp13A	*sakazakii*	Dairy Facility, Environmental	ND	ND
CQ31	*sakazakii*	PIF Facility, Environmental	CsakO:2	4
E788	*sakazakii*	Clinical	CsakO:2	4
Comp48A	*sakazakii*	Dairy Facility, Environmental	CsakO:7	ND
Comp18A	*sakazakii*	Dairy Facility, Environmental	CsakO:7	ND
LR746	*sakazakii*	Food,ISO III powder	CsakO:3	ND
Md5s	*sakazakii*	Fly, *M. domestica*, surface	CsakO:3	4
3Md5g41gF	*sakazakii*	Fly, *M. domestica*, gut	CsakO:2	4
2156.3	*sakazakii*	Clinical,Blood	CsakO:3	4
10Md5g37Fly	*sakazakii*	Fly, *M. domestica*, gut	CsakO:2	4
Md5gD	*sakazakii*	Fly, *M. domestica*, gut	CsakO:2	4
Md1g	*sakazakii*	Fly, *M. domestica*, gut	CsakO:2	ND
CQ118	*E. hormaechei*	PIF Facility, Environmental	ND	ND
Fpul1160	*F. pulveris*	Fruit powder	ND	ND
Fpul601	*F. pulveris*	Fruit powder	ND	ND
Cfreundii576	*C. freundii*	Unknown	ND	ND
Kpneumoniae214	*K. pneumoniae*	Unknown	ND	ND
SalSTM	*S.* Typhimurium	Unknown	ND	ND
CQ119	*E. gormaechei*	PIF Facility, Environmental	ND	ND
CQ125	*C. sedlakii*	PIF Facility, Environmental	ND	ND
Stur508	*S. turicensis*	Fruit powder	ND	ND

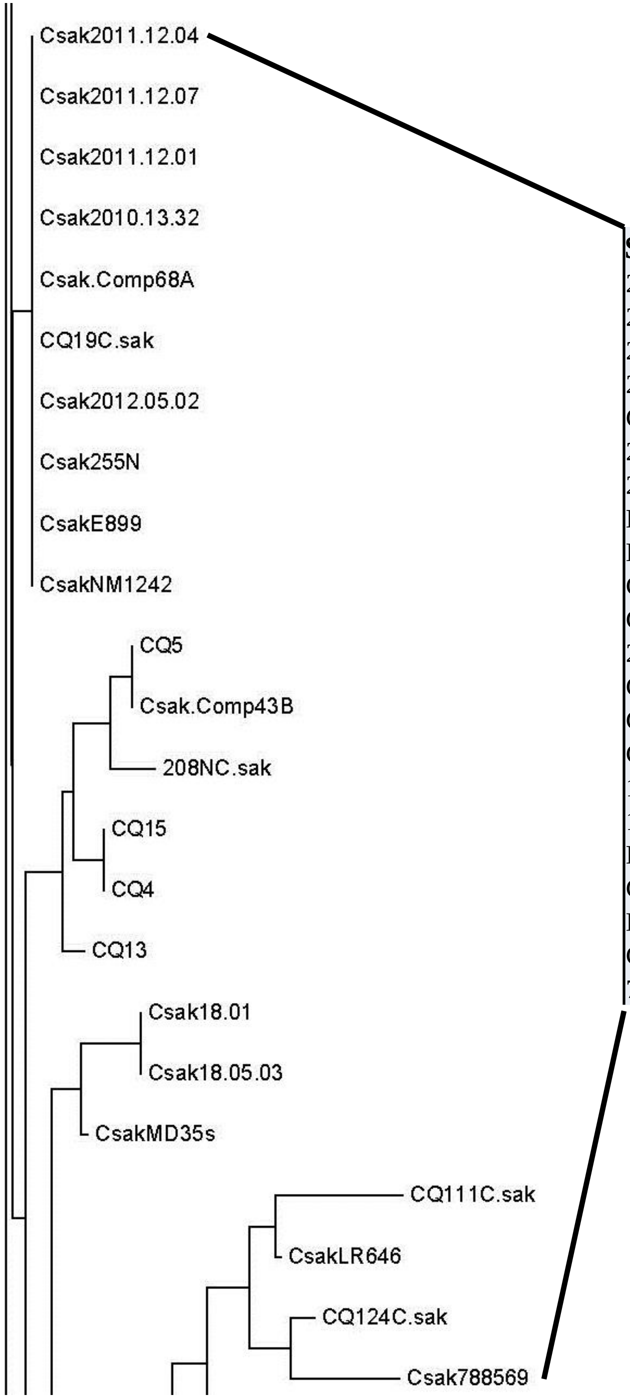

**Strain**	**Species**	**Source**	**Serotype**	**ST**
2011.12.04	*sakazakii*	Food, Powdered infant Formula	CsakO:2	4
2011.12.07	*sakazakii*	Clinical, CSF	CsakO:2	4
2011.12.01	*sakazakii*	Clinical, CSF	CsakO:2	4
2010.13.32	*sakazakii*	Clinical, CSF	CsakO:2	4
CQ19	*sakazakii*	PIF Facility. Environment	CsakO:2	4
2012.05.02	*sakazakii*	Food, PIF, Reconstituted bottle	CsakO:4	4
255N	*sakazakii*	Clinical	CsakO:2	4
E899	*sakazakii*	Clinical	CsakO:2	4
NM1242	*sakazakii*	Clinical, Brain	CsakO:4	4
CQ5	*sakazakii*	PIF Facility, Environment	CsakO:2	4
Comp43B	*sakazakii*	Dairy Facility, Environmental	CsakO:2	ND
208N	*sakazakii*	Clinical	CsakO:2	4
CQ15	*sakazakii*	PIF Facility, Environment	CsakO:2	4
CQ4	*sakazakii*	PIF Facility, Environment	CsakO:2	4
CQ13	*sakazakii*	PIF Facility, Environment	CsakO:2	4
18.01	*sakazakii*	Clinical, Stool	CsakO:1	4
18.05.03	*sakazakii*	Opened PIF container	CsakO:1	8
Md35s	*sakazakii*	Fly, *M. domestica*, surface	ND	8
CQ111	*sakazakii*	PIF Facility, Environment	CsakO:3	4
LR646	*sakazakii*	Food, Soy flour	CsakO:1	ND
CQ124	*sakazakii*	PIF Facility, Environment	CsakO:3	4
788569	*sakazakii*	Food, Siberian Ginseng, (*E sentiocosus*)	CsakO:2	ND

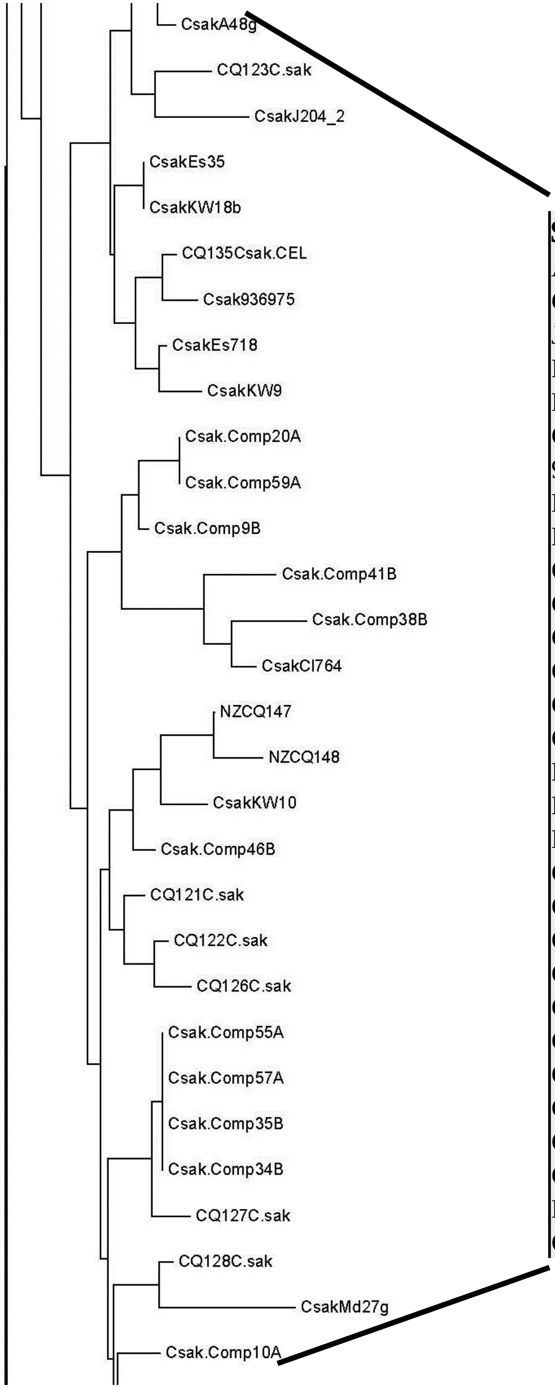

Strain	Species	Source	Serotype	ST
A48g	*sakazakii*	Fly, *Anthimylidae*, gut	ND	221
CQ123	*sakazakii*	PIF Facility, Environmental	CsakO:3	4
J204_2	*sakazakii*	Food, Liquorice	CsakO:7	223
Es35	*sakazakii*	Clinical	CsakO:1	8
KW18b	*sakazakii*	Food, Mushroom	CsakO:1	ND
CQ135	*sakazakii*	PIF Facility, Environmental	CsakO:2	4
936975	*sakazakii*	Unknown	CsakO:2	4
Es718	*sakazakii*	Clinical	CsakO:3	ND
KW9	*sakazakii*	Food, Sorghum	ND	ND
Comp20A	*sakazakii*	Dairy Facility, Environmental	CsakO:2	64
Comp59A	*sakazakii*	Dairy Facility, Environmental	CsakO:2	64
Comp9B	*sakazakii*	Dairy Facility, Environmental	CsakO:2	ND
Comp41B	*sakazakii*	Dairy Facility, Environmental	CsakO:4	ND
Comp38B	*sakazakii*	Dairy Facility, Environmental	CsakO:4	ND
Cl764	*sakazakii*	Clinical	CsakO:4	12
NZCQ147	*sakazakii*	PIF Facility, Environmental	ND	ND
NZCQ148	*sakazakii*	PIF Facility, Environmental	ND	ND
KW10	*sakazakii*	Food, Powdered Pine needles	CsakO:2	ND
Comp46B	*sakazakii*	Dairy Facility, Environmental	CsakO:2	64
CQ121	*sakazakii*	PIF Facility, Environmental	CsakO:3	4
CQ122	*sakazakii*	PIF Facility, Environmental	CsakO:3	4
CQ126	*sakazakii*	PIF Facility, Environmental	CsakO:3	4
Comp55A	*sakazakii*	Dairy Facility, Environmental	CsakO:2	ND
Comp57A	*sakazakii*	Dairy Facility, Environmental	CsakO:2	64
Comp35B	*sakazakii*	Dairy Facility, Environmental	CsakO:2	ND
Comp34B	*sakazakii*	Dairy Facility, Environmental	CsakO:2	ND
CQ127	*sakazakii*	PIF Facility, Environmental	CsakO:3	4
CQ128	*sakazakii*	PIF Facility, Environmental	CsakO:3	4
Md27g	*sakazakii*	Fly, *M. domestica*, gut	ND	7
Comp10a	*sakazakii*	Dairy Facility, Environmental	CsakO:4	ND

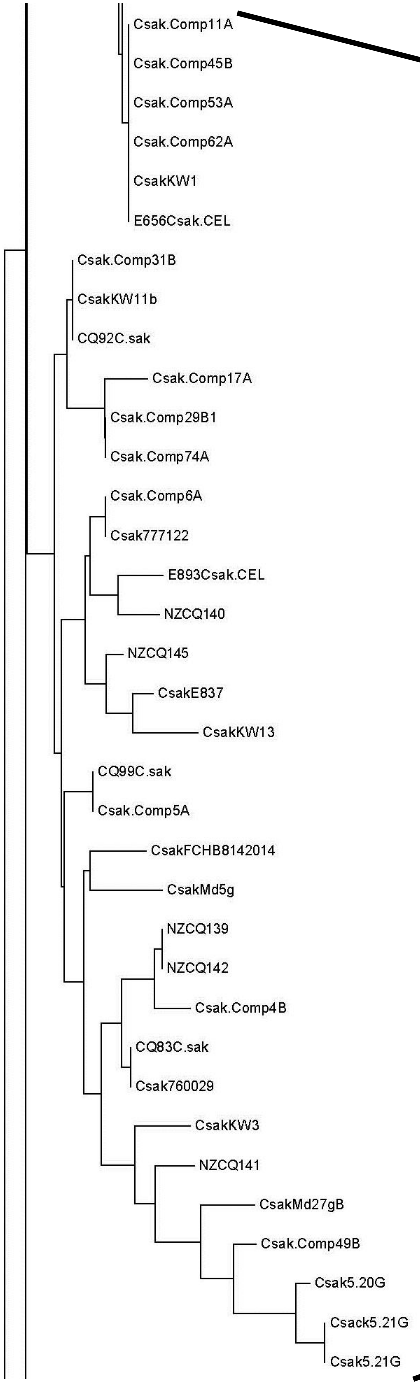

Strain	Species	Source	Serotype	ST
Comp11a	*sakazakii*	Dairy Facility, Environmental	CsakO:2	64
Comp45b	*sakazakii*	Dairy Facility, Environmental	CsakO:2	64
Comp53a	*sakazakii*	Dairy Facility, Environmental	CsakO:2	64
Comp62a	*sakazakii*	Dairy Facility, Environmental	CsakO:2	1
KW1	*sakazakii*	Food, Barely	ND	ND
E656	*sakazakii*	Clinical	CsakO:1	8
Comp31b	*sakazakii*	Dairy Facility, Environmental	CsakO:4	ND
KW11b	*sakazakii*	Food, Black Bean	CsakO:4	ND
CQ92C	*sakazakii*	PIF Facility, Environmental	CsakO:4	40
Comp17a	*sakazakii*	Dairy Facility, Environmental	CsakO:4	ND
Comp29b1	*sakazakii*	Dairy Facility, Environmental	CsakO:4	40
Comp74a	*sakazakii*	Dairy Facility, Environmental	CsakO:4	1
Comp6a	*sakazakii*	Dairy Facility, Environmental	CsakO:4	ND
777122	*sakazakii*	Food, Hulled Seseme Seed	CsakO:4	ND
E893	*sakazakii*	Clinical	CsakO:2	4
NZCQ140	*sakazakii*	PIF Facility, Environmental	ND	ND
NZXQ145	*sakazakii*	PIF Facility, Environmental	ND	ND
E837	*sakazakii*	Clinical	CsakO:2	ND
KW13	*sakazakii*	Food, Dried Garlic	CsakO:1	ND
CQ99	*sakazakii*	PIF Facility, Environmental	CsakO:4	6
Comp5a	*sakazakii*	Dairy Facility, Environmental	CsakO:2	ND
FCHB8142014	*sakazakii*	Chicken feed	CsakO:4	ND
Md5g	*sakazakii*	Fly, *M. domestica*, gut	CsakO:2	4
NZCQ139	*sakazakii*	PIF Facility, Environmental	ND	13
NZCQ142	*sakazakii*	PIF Facility, Environmental	ND	ND
Comp4b	*sakazakii*	Dairy Facility, Environmental	CsakO:2	ND
CQ83	*sakazakii*	PIF Facility, Environmental	CsakO:4	40
760029	*sakazakii*	Food, Whole grain, Corn	CsakO:4	ND
KW3	*sakazakii*	Food, Dried Hot Pepper	CsakO:4	ND
NZCQ141	*sakazakii*	PIF Facility, Environmental	ND	42
Md27gB	*sakazakii*	Fly, *M. domestica*, gut	ND	7
Comp49b	*sakazakii*	Dairy Facility, Environmental	CsakO:2	64
5.20g	*sakazakii*	Food, Nuts	CsakO:2	ND
5.21g	*sakazakii*	Food, Nuts	CsakO:2	ND

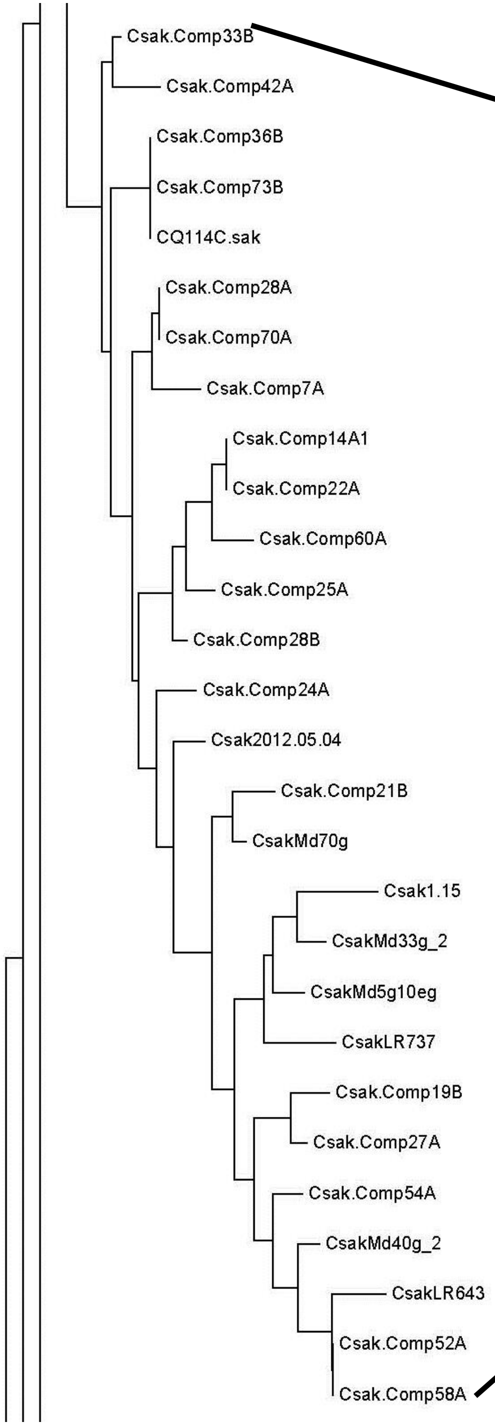

Strain	Species	Source	Serotype	ST
Comp33b	*sakazakii*	Dairy Facility, Environmental	CsakO:2	ND
Comp42a	*sakazakii*	Dairy Facility, Environmental	CsakO:2	ND
Comp36b	*sakazakii*	Dairy Facility, Environmental	CsakO:2	ND
Comp73b	*sakazakii*	Dairy Facility, Environmental	CsakO3	ND
CQ114	*sakazakii*	PIF Facility, Environmental	CsakO:2	31
Comp28a	*sakazakii*	Dairy Facility, Environmental	CsakO:2	ND
Comp70a	*sakazakii*	Dairy Facility, Environmental	CsakO:2	ND
Comp7a	*sakazakii*	Dairy Facility, Environmental	CsakO:4	ND
Comp12a1	*sakazakii*	Dairy Facility, Environmental	ND	ND
Comp22a	*sakazakii*	Dairy Facility, Environmental	CsakO:2	ND
Comp60a	*sakazakii*	Dairy Facility, Environmental	CsakO:7	ND
Comp25a	*sakazakii*	Dairy Facility, Environmental	CsakO:2	ND
Comp28b	*sakazakii*	Dairy Facility, Environmental	CsakO:2	ND
Comp24a	*sakazakii*	Dairy Facility, Environmental	CsakO:2	ND
2012.05.04	*sakazakii*	Food, water	CsakO:4	4
Comp21b	*sakazakii*	Dairy Facility, Environmental	CsakO:1	ND
Md70g	*sakazakii*	Fly, *M. domestica*, gut	CsakO:2	ND
1.15	*sakazakii*	Food, Nuts	CsakO:2	ND
Md33g_2	*sakazakii*	Fly, *M. domestica*, gut	CsakO:1	8
Md5g10eg	*sakazakii*	Fly, *M. domestica*, gut	CsakO:2	4
LR737	*sakazakii*	Food, Org Hi PDI Flour	CsakO:3	ND
Comp19b	*sakazakii*	Dairy Facility, Environmental	CsakO:2	64
Comp27a	*sakazakii*	Dairy Facility, Environmental	CsakO:1	ND
Comp54a	*sakazakii*	Dairy Facility, Environmental	CsakO:2	64
Md40g_2	*sakazakii*	Fly, *M. domestica*, gut	CsakO:2	ND
LR643	*sakazakii*	Food, Soy flour	CsakO:3	ND
Comp52a	*sakazakii*	Dairy Facility, Environmental	CsakO:1	ND
Comp58a	*sakazakii*	Dairy Facility, Environmental	CsakO:1	ND

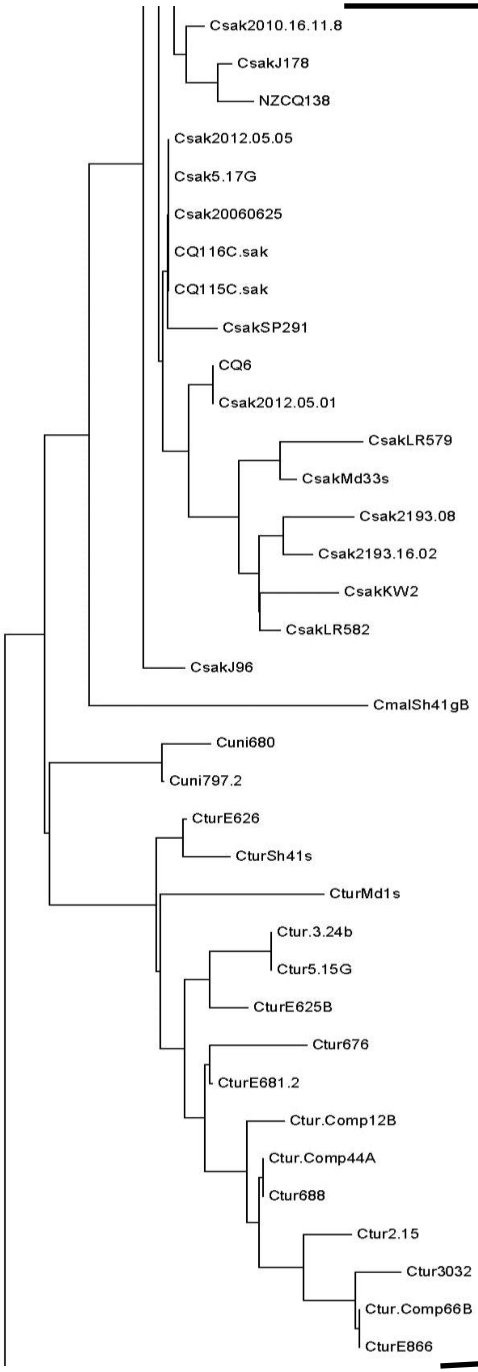

Strain	Species	Source	Serotype	ST
2010.16.11.8	*sakazakii*	Environmental, Baby Pacifer	CsakO:2	4
J178	*sakazakii*	Food, Chamomile, Spice	CsakO:3	ND
NZCQ138	*sakazakii*	PIF Facility, Environmental	ND	4
2012.05.05	*sakazakii*	Clinical, CSF	CsakO:4	4
5.17G	*sakazakii*	Food, Nuts	CsakO:1	ND
20060625	*sakazakii*	Clinical	CsakO:2	4
CQ116	*sakazakii*	PIF Facility, Environmental	CsakO:2	31
CQ115	*sakazakii*	PIF Facility, Environmental	CsakO:2	31
SP291	*sakazakii*	PIF Facility, Environmental	CsakO:2	4
CQ6	*sakazakii*	PIF Facility, Environmental	CsakO:2	4
2012.05.01	*sakazakii*	Food, PIF	CsakO:4	4
LR579	*sakazakii*	Environmental	CsakO:3	ND
Md33S	*sakazakii*	Fly, *M. domestica*, surface	CsakO:1	8
2193.08	*sakazakii*	Food, Nursery Water	CsakO:1	8
2193.16.02	*sakazakii*	Food, Nursery Water	CsakO:1	8
KW2	*sakazakii*	Food, Dried Filefish	CsakO:1	ND
LR582	*sakazakii*	Environmental	CsakO:4	ND
J96	*sakazakii*	Food, Fennel, Spice	CsakO:3	ND
680	*universalis*	Food	ND	ND
797.2	*universalis*	Environmental, water	CuniO:1	54
E626	*turicensis*	Food, Infant food	CturO:3	ND
Sh41S	*turicensis*	Fly, *Sarcophaga haemorrhoidalis*, surface	ND	ND
Md1S	*turicensis*	Fly, *M. domestica*, surface	ND	7
3.24B	*turicensis*	Food, Nuts	ND	ND
5.15G	*turicensis*	Food, Nuts	ND	ND
E625B	*turicensis*	Food, Infant food	CturO:3	ND
676	*turicensis*	Food	CturO:3	ND
E681.2	*turicensis*	Food, Infant food	CturO:3	ND
Comp12B	*turicensis*	Dairy Facility, Environmental	ND	ND
Comp44A	*turicensis*	Dairy Facility, Environmental	ND	ND
688	*turicensis*	Food	ND	ND
2.15	*turicensis*	Food, Nuts	ND	ND
3032	*turicensis*	Clinical, Blood	CturO:1	19
Comp66b	*turicensis*	Dairy Facility, Environmental	CturO1	ND

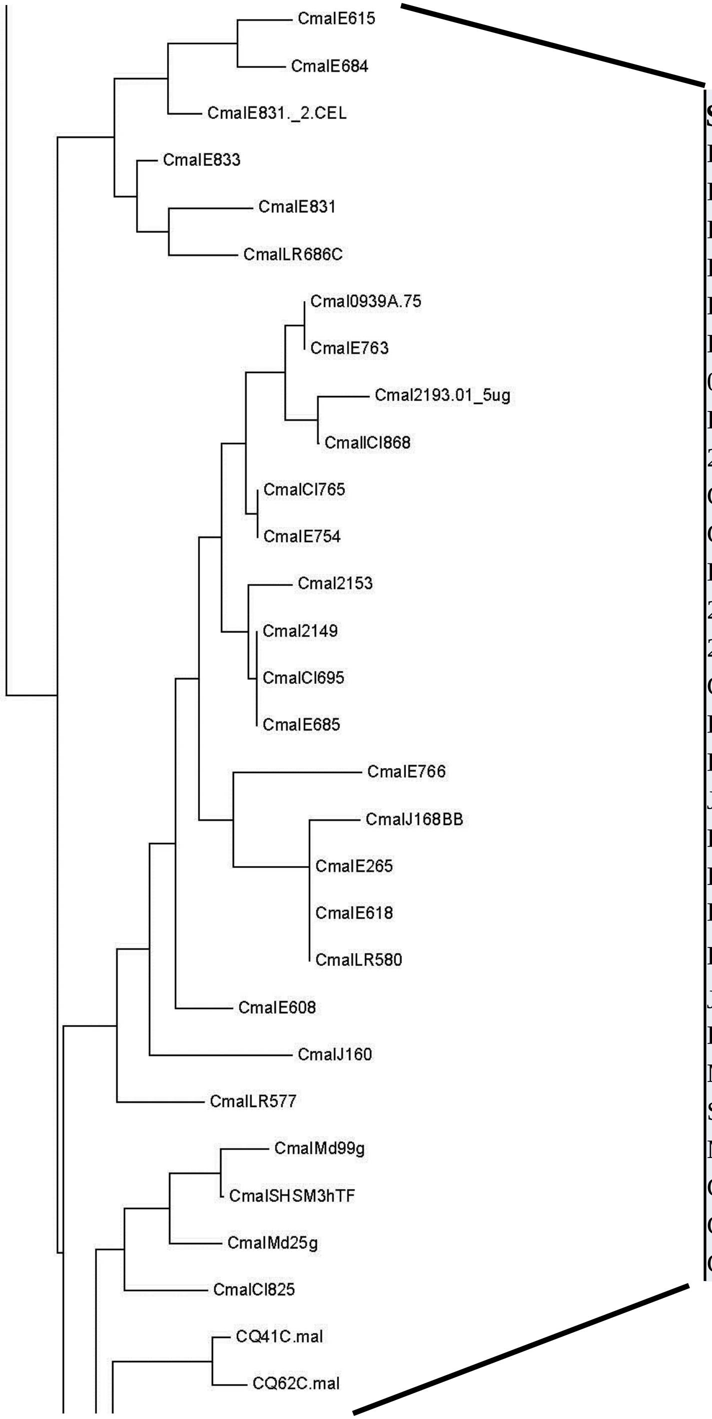

Strain	Species	Source	Serotype	ST
E615	*malonaticus*	Clinical	CmalO:1	ND
E684	*malonaticus*	Food	CmalO:1	ND
E831._2	*malonaticus*	Clinical	CmalO:1	60
E833	*malonaticus*	Clinical	CmalO:1	60
E831	*malonaticus*	Clinical	CmalO:1	60
LR686	*malonaticus*	Food, PIF ingredient	CmalO:1	ND
0939A.75	*malonaticus*	Clinical, Nose	CmalO:2	ND
E763	*malonaticus*	Clinical	CmalO:2	ND
2193.01_5	*malonaticus*	Clinical, Blood	CmalO:1	307
CI868	*malonaticus*	Clinical	CmalO:2	ND
CI765	*malonaticus*	Clinical	CmalO:2	ND
E754	*malonaticus*	Clinical	CmalO:2	ND
2153	*malonaticus*	Clinical, Blood	CmalO:2	ND
2149	*malonaticus*	Clinical, CSF	CmalO:2	ND
CI695	*malonaticus*	Food, Infant food	CmalO:2	ND
E685	*malonaticus*	Food	CmalO:2	ND
E766	*malonaticus*	Food, Milk powder (MP)	CmalO:2	ND
J168BB	*malonaticus*	Food, Spices	CmalO:2	ND
E265	*malonaticus*	Food, MP	CmalO:2	ND
E618	*malonaticus*	Clinical	CmalO:2	ND
LR580	*malonaticus*	Environmental	CmalO:2	ND
E608	*malonaticus*	Clinical	CmalO:2	ND
J160	*malonaticus*	Environmental, Vacuum dust	CmalO:2	ND
LR577	*malonaticus*	Environmental	CmalO:2	ND
Md99g	*malonaticus*	Fly, *M. domestica*, gut	CmalO:1	7
SHSM3hTF	*malonaticus*	Environmental, Tomato field	CmalO:1	ND
Md25g	*malonaticus*	Fly, *M. domestica*, gut	CmalO:2	7
CI825	*malonaticus*	Clinical, Breast abscess	CmalO:2	7
CQ41	*malonaticus*	PIF Facility, Environmental	CmalO:2	129
CQ62	*malonaticus*	PIF Facility, Environmental	CmalO:2	ND

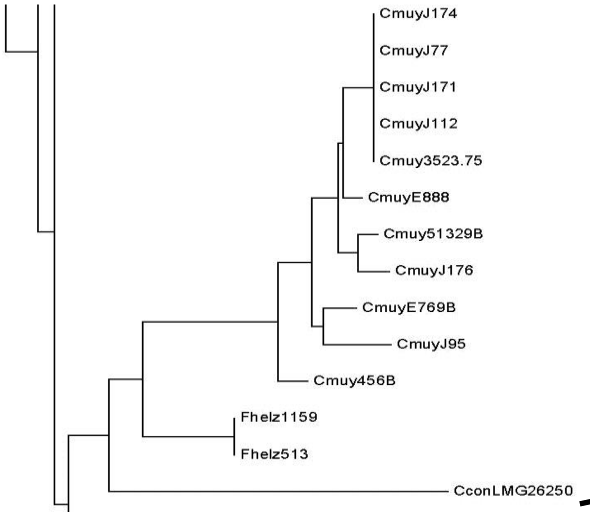

**Strain**	**Species**	**Source**	**Serotype**	**ST**
J174	*muytjensii*	Food, Anise, Spice	CmuyO:1	ND
J77	*muytjensii*	Food, Anise, Spice	CmuyO:2	ND
J171	*muytjensii*	Fennel, Food, Spice	ND	ND
J112	*muytjensii*	Food, Liquorice	ND	ND
3523.75	*muytjensii*	Clinical, Bone marrow	CmuyO:2	5
E888	*muytjensii*	Food, MP	ND	ND
51329B	*muytjensii*	Unknown	CmuyO:2	81
J176	*muytjensii*	Food, Thyme, Spice	ND	ND
E769B	*muytjensii*	Food, MP	CmuyO:1	ND
J95	*muytjensii*	Food, Anise, Spice	ND	ND
456B	*muytjensii*	Unknown	CmuyO:2	ND
Fhelz1159	*F.helveticus*	Fruit powder	ND	ND
Fhelz513	*F.helveticus*	Fruit powder	ND	ND
LMG26250	*condimenti*	Food, spiced sausage	ND	98

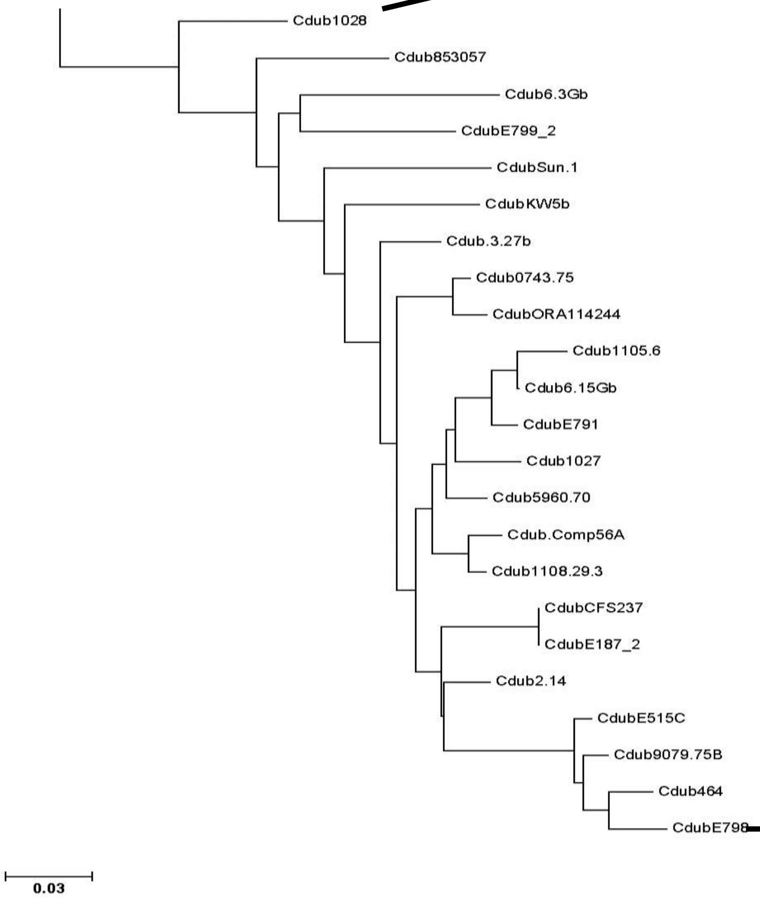

Strain	Species	Source	Serotype	ST
1028	*dublinensis*	MP Facility, Environmental	CdubO:1	ND
853057	*dublinensis*	Fresh Basil	ND	ND
6.3Gb	*dublinensis*	Food, Nuts	CdubO:1	ND
E799_2	*dublinensis*	Unknown	CdubO:2	ND
Sun.1	*dublinensis*	Food, Taro	ND	ND
KW5b	*dublinensis*	Food, Black Rice	CdubO:1	ND
3.27B	*dublinensis*	Food, Nuts	CdubO:1	ND
743.75	*dublinensis*	Clinical, Foot wound	CdubO:1	41
ORA114244	*dublinensis*	Oregano	CduO:2	ND
1105.6	*dublinensis*	Vegetable	ND	ND
6.15Gb	*dublinensis*	Food, Nuts	CdubO:2	ND
E791	*dublinensis*	unknown	CdubO:1	ND
1027	*dublinensis*	MP facility, Environmental	ND	ND
5960.7	*dublinensis*	Clinical, Blood	ND	5
Comp56a	*dublinensis*	Dairy Facility, Environmental	CdubO:1	ND
1108.29.3	*dublinensis*	Food,Vegetable	CdubO:1	ND
CFS237	*dublinensis*	MP facility, Environmental	CdubO:1	106
E187_2	*dublinensis*	Unknown	CdubO:1	106
2.14	*dublinensis*	Food, Nuts	CdubO:1	ND
E515C	*dublinensis*	Environmental, Water fountain	CdubO:2	80
9079.75B	*dublinensis*	Unknown	CdubO:2	36
464	*dublinensis*	MP facility, Environmental	CdubO:1	8
E798	*dublinensis*	Unknown	CdubO:2	ND

Supplemental Figure 1. Hierarchical clustering of RMA-summarized microarray data employing a database of over 240 *Cronobacter* and phylogenetically-related strains which were generated from the gene difference matrix of the 59 OMP genes captured on the pan genomic *Cronobacter* DNA microarray. The microarray experimental protocol as described by Tall et al. (2015) was used for the interrogation of the strains and for the analysis. The phylogenetic tree illustrates that the *Cronobacter* microarray could clearly separate the seven species of *Cronobacter*, with each species forming its own distinct cluster. The tree was generated using the Neighbor-joining method using MEGA6 (2013) and 1000 bootstrap replicates. The scale bar for Supplemental Figure 1 represents 0.03 base substitutions.

Tall, B. D., Gangiredla, J. Gopinath, G. R., Yan, Q. Q., Chase, H. R., Lee, B., Hwang, S., Trach, L., Park, E., Yoo, Y.J., Chung, T., Jackson, S. A., Patel,. R., Sathyamoorthy, V., Pava-Ripoll, M., M. L. Kotewicz, M. L., Carter L., Iversen, C., Pagotto, F., Stephan, R., Lehner, A., Fanning, S., and Grim, C. J. 2015. Development of a custom-designed, pan genomic DNA microarray to characterize strain-level diversity among *Cronobacter* spp. Front Pediatr **2015**, 3, 66. doi: 10.3389/fped.2015.00036. eCollection 2015.

Tamura K, Stecher G, Peterson D, Filipski A, and Kumar S (2013) MEGA6: Molecular Evolutionary Genetics Analysis Version 6.0. Molecular Biology and Evolution 30:2725-2729
